# Immune-Based Biomarkers as Predictors of Mortality in ECMO Therapy for Severe COVID-19 ARDS: Insights from a Retrospective Study

**DOI:** 10.3390/ijms27010390

**Published:** 2025-12-30

**Authors:** Rosalia Busà, Giovanna Panarello, Alessia Gallo, Vitale Miceli, Salvatore Castelbuono, Maria Concetta Sorrentino, Giandomenico Amico, Claudia Carcione, Giovanna Russelli, Nicola Cuscino, Monica Miele, Francesca Timoneri, Mariangela Di Bella, Giovanni Zito, Floriana Barbera, Ester Badami, Anna Maria Corsale, Mojtaba Shekarkar Azgomi, Pier Giulio Conaldi, Cirino Botta, Matteo Bulati

**Affiliations:** 1 IRCCS ISMETT, 90127 Palermo, Italy; 2 Department of Engineering, University of Palermo, 90128 Palermo, Italy; 3 Ri.MED Foundation, 90133 Palermo, Italy; 4 Department of Health Promotion, Mother and Child Care, Internal Medicine and Medical Specialties, University of Palermo, 90127 Palermo, Italy; 5 Department of Biomedicine, Neuroscience and Advanced Diagnostic Diagnostic (BIND), University of Palermo, 90133 Palermo, Italy

**Keywords:** ECMO, COVID-19, ICU, ARDS, T cell exhaustion, cytokinome

## Abstract

Extracorporeal membrane oxygenation (ECMO) is a vital intervention for patients with severe respiratory failure, particularly in unresponsive acute respiratory distress syndrome (ARDS) cases. However, patient selection for ECMO remains a significant challenge. This study aims to identify novel immune-based biomarkers to improve eligibility assessment and predict outcomes in critically ill COVID-19 patients undergoing ECMO. This monocentric observational retrospective cohort study included 80 patients with severe COVID-19-related pneumonia who required ECMO support due to unresponsive ARDS. The patients were admitted to the intensive care unit (ICU) of IRCCS-ISMETT Hospital between September 2020 and April 2021, before the availability of COVID-19 vaccines. All patients were infected with the original SARS-CoV-2 Wuhan strain. Using machine learning approaches, the study analyzed clinical and laboratory data, cytokine levels, RNA sequencing (RNA-seq), and immune cell profiles collected within two days of hospitalization. The analysis identified a 5.56-fold increased mortality risk in patients presenting with a combination of immune factors: a T cell exhaustion profile, low interferon-alpha (IFNα) levels, and high calprotectin levels. These immune markers were strongly associated with poorer outcomes in patients undergoing ECMO. Our findings highlight the critical role of immune profiling in ECMO patient selection and outcome prediction. Incorporating immune-based biomarkers into clinical assessments may enhance the evaluation of ECMO eligibility and guide treatment decisions, ultimately improving patient outcomes.

## 1. Introduction

Extracorporeal membrane oxygenation (ECMO) is a vital intervention for severe respiratory failure, including acute respiratory distress syndrome (ARDS), which results from lung inflammation [1]. The successful use of ECMO was demonstrated during the H1N1 and COVID-19 pandemics, with several studies highlighting that patient outcomes hinge on factors such as age, gender, chronic lung disease, symptom duration, mechanical ventilation, and the experience of the skills center in ECMO patient management [1,2,3,4,5,6]. The Extracorporeal Life Support Organization (ELSO) recommends considering ECMO when the mortality risk is around 50%, deeming it nearly indispensable at an 80% mortality risk [7]. Additionally, the cost/benefit ratio is pivotal for ECMO eligibility due to the associated higher healthcare costs [8]. Despite the crucial medical and economic importance of precise identification of ECMO-eligible patients, a comprehensive scoring system considering biological and clinical parameters is still lacking. Decisions regarding ECMO support are tailored to individual cases, accounting for overall patient condition, prognosis, and immune system competence, which are especially critical in severe COVID-19 cases leading to ARDS [9,10]. Here we present a monocentric observational retrospective study aimed at identifying new and more detailed immunological biomarkers as an additional tool to already known clinical predictors [11] useful for ECMO patient management. The study involved 80 patients with severe COVID-19-related pneumonia, admitted to the intensive care unit (ICU) of IRCCS-ISMETT Hospital, who required ECMO support due to unresponsive ARDS. The patients were managed between September 2020 and April 2021, before the advent of COVID-19 vaccines, and were infected with the original SARS-CoV-2 Wuhan strain. The data obtained from a deep immune profile characterization of our ECMO cohort (multi-parametric flow cytometry, cytokine array, RNA-seq, and clinical data) were fed to a machine-learning model, identifying three major predictor factors that seem to be related to worse outcomes. Particularly, we found an increased death risk of 5.56-fold in patients who simultaneously showed a T cell exhaustion profile, as already described by Ulakcsai et al. [12,13], low levels of IFNα, and high levels of calprotectin. In critical conditions, the importance of prompt ECMO eligibility is fundamental for the patient’s outcome. For that reason, the identification of additional and more accurate biomarkers, which can be easily detected in blood samples using non-invasive and repeatable methods, together with current clinical predictors, could aid physicians in making timely decisions. Based on our findings, incorporating the assessment of T cell status and the dosage of IFNα and calprotectin could improve ECMO eligibility evaluation and treatment guidance. However, this study was conducted in a small cohort of ECMO patients and therefore needs to be validated in a larger prospective ECMO cohort.

## 2. Results

### 2.1. Patient Characteristics at Baseline

Between September 2020 and April 2021, a total of 80 ECMO patients (mean age ± SD = 50.2 ± 8.8 years; 61 male) affected with COVID-19-related pneumonia, complicated by respiratory failure with severe ARDS unresponsive to conventional man oeuvres, were admitted to our institute (IRCCS-ISMETT) (Table 1).

**Figure 1:**
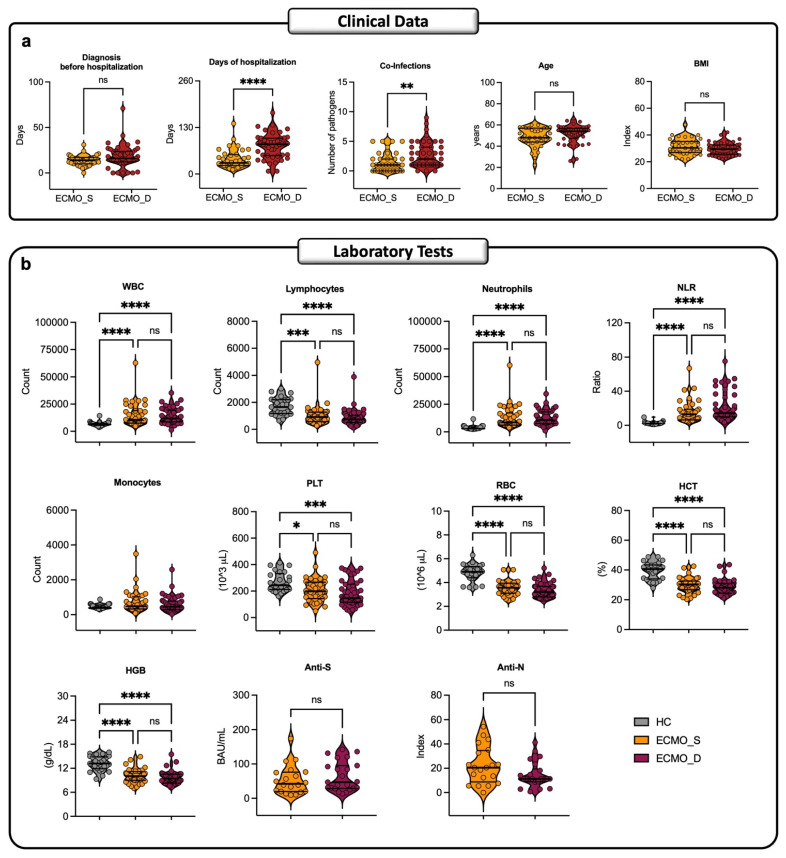
Study cohort characteristics at baseline. (**a**) The clinical data include the median values of the number of days before hospitalization and the number of days of hospitalization at discharge or time of death, the number of co-infections detected during the hospitalization, age, and body mass index (BMI) in ECMO-survived (ECMO_S n = 36) and ECMO-deceased patients (ECMO_D n = 44). The significance was determined using a Mann–Whitney U-test (two-sided). (**b**) The laboratory tests were done on blood, serum, or plasma from both ECMO cohorts (ECMO_S and ECMO_D), and when possible, compared to the healthy control cohort (HC), on biological markers. The laboratory tests include the median values of anti-S (BAU/mL) and N (Index) IgG, the median count of white blood cells (WBC), lymphocytes, neutrophils, monocytes, the neutrophil-to-lymphocyte ratio (NLR), levels of platelets (PLT), red blood cells (RBC), hemoglobin (HGB) and, finally, the median percentage of hematocrit (HCT). The significance was determined using a Mann–Whitney U-test (two-sided) and a Kruskal–Wallis test with Dunn’s multiple comparisons. * *p* < 0.05, ** *p* < 0.01, *** *p* < 0.001. **** *p* < 0.0001.

Among the 80 ECMO patients included in this study, 44 died, while 36 survived. Figure 1a shows no difference in the days of diagnosis before hospitalization, age, and BMI between the two groups. We found statistical differences in terms of days of hospitalization (*p* < 0.0001) and number of co-infections (*p* = 0.0032), which were both higher in ECMO_D compared to ECMO_S patients. Biochemical and hematological laboratory parameters, shown in Figure 1b, revealed that WBC, lymphocytes, neutrophils, NLR, PLT, RBC, HCT, and HGB levels were higher in both ECMO groups compared to the HC cohort, and no difference was observed between ECMO_S and ECMO_D patients. Conversely, we did not find any statistical difference in monocyte count, anti-S levels, or anti/N antibody levels, nor among ECMO or HC cohorts.

### 2.2. Cytokinome Profile Evaluation in Critical COVID-19 ICU Patients

**Figure 2:**
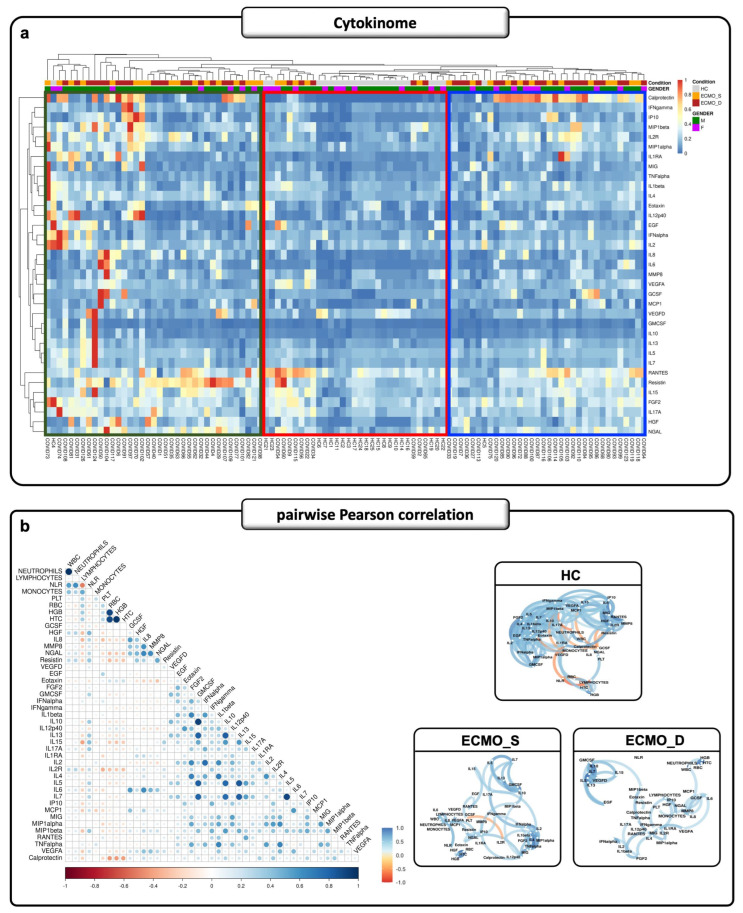
The cytokinome signature associated with fatal outcomes in ECMO patients. (**a**) The clustered heatmap shows the cytokinome dataset of 35 circulation cytokines/chemokines in the baseline plasma samples from 23 HCs (grey) and 80 ECMO patients, labeled by gender (F and M as purple and green, respectively), including 36 ECMO_S (orange) and 44 ECMO_D (red) patients. The cluster analysis reveals three distinct cytokine signatures: S1 (green), S2 (red), and S3 (blue). The cytokine expressions were corrected and scaled to a range between 0 (blue) and 1 (red). (**b**) Pairwise Pearson correlation matrix among all 35 circulating cytokines and 9 different clinical features for all studied groups. Corr-plot (on the left panel) and network plots (on the right panel) show positive (blue) and negative (red) correlations. For a better overview, only significant differences (*p* < 0.05) are displayed. F: female; M: male; HCs: healthy control; ECMO_S: ECMO survived; ECMO_D: ECMO deceased.

The cluster analysis revealed (Figure 2a) three distinct cytokine signatures: S1 (green), S2 (red), and S3 (blue). The S1 signature, which includes 46% of ECMO_S patients (17/37), 51% of ECMO_D patients (19/37), and only 3% of HCs (1/37), showed an overall higher cytokine level compared to S2 and S3. The S2 signature includes mostly HCs, at 68% (21/31), but also 16% of ECMO_S (5/31) and 16% of ECMO_D patients (5/31), while S3 includes only 3% of HCs (1/34) as well as 38% (13/34) and 59% (20/34) of ECMO_S and ECMO_D patients, respectively. We observed higher levels of RANTES, resistin, HGF, NGAL, IL2R, IL15, FGF2, IL17A, and calprotectin in ECMO_D compared to ECMO_S patients and HCs, suggesting a specific cytokine signature associated with a worse state of disease and patient condition. Figure 2b highlights the relationship between each cytokine and clinical features, with multiple significant direct and indirect correlations between these parameters. Particularly, 16 of 35 cytokines (HGF, MMP8, NGAL, resistin, IL2R, IL8, GM-CSF, IL10, IL13, IL15, IL17A, IL7, MCP-1, EGF, eotaxin, RANTES) were highly positively correlated with WBC, neutrophils, NLR, and PLT, suggesting the activation of myeloid-driven immune responses. In contrast, we observed a highly negative correlation in 13 of 35 cytokines (HGF, IL8, NGAL, resistin, IL13, IL15, IL1RA, IL2R, IL6, IP10, MCP-1, MIP-1β, and VEGFA) with lymphocytes, suggesting a potential suppression of lymphocyte activity in patients with elevated levels of these cytokines [14]. Finally, RBC, HGB, and HCT were negatively correlated with 5 of 35 cytokines (IL8, NGAL, resistin, IL2R, and calprotectin). High levels of these cytokines in COVID-19 are related to negative effects on the RBC ultrastructure, which can induce eryptosis [15]. Additionally, the network plots revealed a reduction in the number of significant correlations between cytokines and clinical features in the ECMO groups compared to HCs. Interestingly, the disappearance of coordinated activation patterns was increased in the ECMO group, especially in ECMO_D patients, in which we found a consistent reduction in the number of significant correlation network lines (Figure 2b and Supplementary Figure S3).

### 2.3. Cytokine Production in ECMO Patients Also Depends on Inflamed Peripheral Blood Cells

CIBERSORTx deconvolution analysis confirmed the distribution of the different populations as determined by flow cytometry, thus supporting the representativeness of the samples (Supplementary Figure S4a, left panel). This behavior is also evidenced in the correlation heatmap, in which we observed strong negative correlations between neutrophils and monocytes, CD4 and CD8 T lymphocytes, and NK cells (Supplementary Figure S4a, right panel). Interestingly, in these patients, we found increased mRNA expressions of CD274 (PD-L1) and FCGR1A (CD64) genes (Supplementary Figure S4b), which allowed us to hypothesize an immature-like/immunosuppressive phenotype of increased circulating neutrophils [16]. In Supplementary Figure S4c, we identified a direct correlation between plasma levels and mRNA expression coming from whole blood cells for a group of cytokines, including HGF, IL-8, NGAL, MMP8, resistin, calprotectin, and VEGFA. These data support the hypothesis that these molecules are produced in the peripheral blood (neutrophil activation signature) [17] in both surviving and deceased ECMO patients compared to HCs. Unfortunately, due to the lack of lung samples, we could only speculate that the increase of these cytokines may be directly related to lung injury, with worse outcomes in COVID-19, as reported elsewhere [17,18,19,20]. Finally, in ECMO patients, we found the well-established impaired production of IFNα [21,22], combined with the down-regulated expression of IFNA1 mRNA and of its key transcription factor IRF3 (Supplementary Figure S4d).

### 2.4. Exhausted T Cell Immune Signature in ECMO-Supported Patients

**Figure 3:**
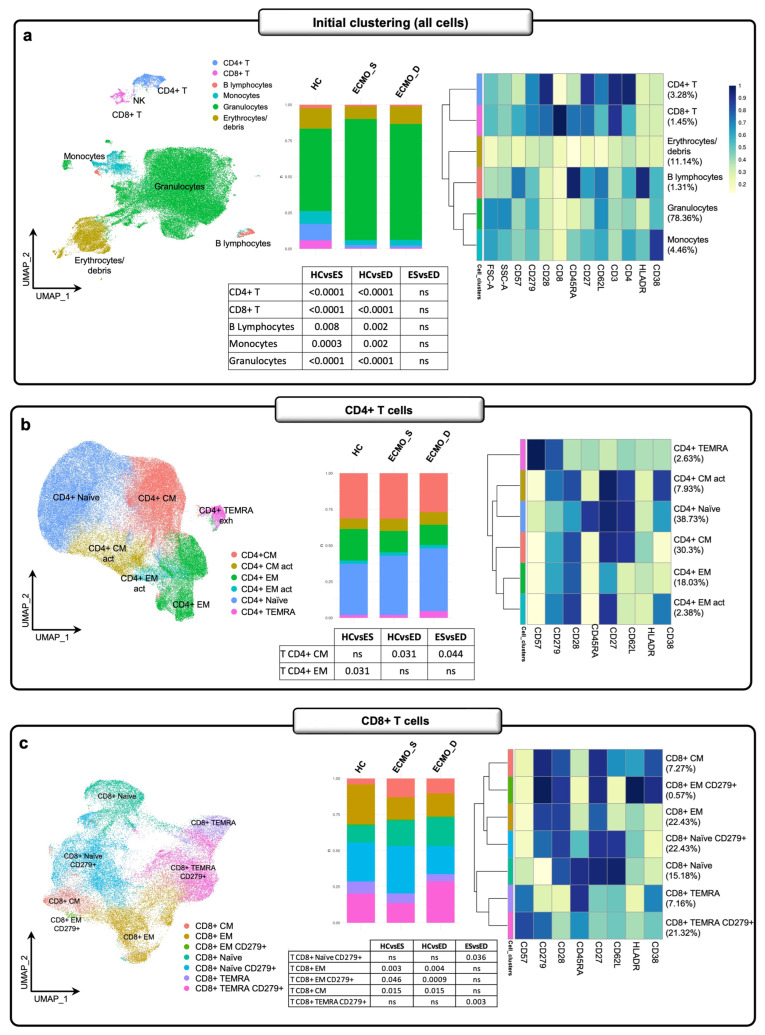
Flow cytometry suggests an exhausted T cell immune signature in ECMO-supported patients. (**a**) UMAP density plot shows the distribution of the six major subpopulations (CD4+ and CD8+ T lymphocytes, B lymphocytes, monocytes, granulocytes, and erythrocytes/debris) identified by flow cytometry on the peripheral blood of 26 ECMO_S and 28 ECMO_D patients, obtained within two days of hospital admission, and 14 HCs. The bar graph indicates the proportion of each cell subset (% of cells) in the whole blood of the three different subgroups, and the table below shows the statistical significance. The canonical markers used to define the six major cell types are shown in the right panel. (**b**) The proportion of total CD4^+^ T cell subpopulations (CD4^+^ CM, CD4^+^ CM act, CD4^+^ EM, CD4^+^ EM act, CD4^+^ naïve, and CD4^+^ TEMRA) from each studied group. The panel on the left shows the UMAP density plot. The bar graph and the table alongside show the proportion of each cell subset (% of cells) and the statistical significance of each CD4^+^ T cell subpopulation in the peripheral blood of the three studied cohorts. The right panel shows the markers used to discriminate CD4^+^ T subpopulations. (**c**) The proportion of total CD8^+^ T cell subpopulations (CD8^+^ CM, CD8^+^ EM, CD8^+^ EM CD279^+^, naïve CD8^+^ CD279^+^, CD8^+^ TEMRA, and CD8^+^ TEMRA CD279^+^) in the studied cohorts. The panel on the left shows the UMAP density plot. The bar graph and the table alongside show the proportion of each cell subset (% of cells) and the statistical significance of each CD8 T^+^ cell subpopulation in the peripheral blood of the three studied groups. The right panel shows the markers used to discriminate CD8^+^ T subpopulations. Significances between groups were determined using the Wilcox test. HC: healthy control; ECMO_S: ECMO survived; ECMO_D: ECMO deceased; CD4^+^ T CM: lymphocytes T CD4^+^ central memory; CD4^+^ T CM act: lymphocytes T CD4^+^ central memory activated; CD4^+^ T EM: lymphocytes T CD4^+^ effector memory; CD4^+^ T EM act: lymphocytes T CD4^+^ effector memory activated; CD8^+^ T CM: lymphocytes T CD8^+^ central memory; CD8^+^ T EM: lymphocytes T CD8^+^ effector memory.

By high-dimensional flow cytometry, we evaluated the peripheral immune assets in 54 patients (26 ECMO_S and 28 ECMO_D) and 14 HCs, using a recently developed bioinformatic pipeline for semi-supervised analysis of flow cytometry data [23,24]. In Figure 3a, we show the significant reduction of CD4^+^ T, CD8^+^ T, B lymphocytes, and monocytes and, conversely, a broad increase of granulocytes in both groups of patients compared to HCs. Moreover, we additionally clustered CD4^+^ T, CD8^+^ T, mucosal-associated invariant T cells (MAIT), Th17, Tγδ, NK/NKT, and B lymphocytes. As depicted in Figure 3b, we found a significant reduction in CD4^+^ central memory (CM) in ECMO patients compared to HCs (*p* = 0.031), as well as in ECMO_D vs. ECMO_S patients (*p* = 0.044). Conversely, we found significant reductions in CD4^+^ effector memory (EM) only in ECMO_S patients compared to HCs (*p* = 0.031). Concerning CD8^+^ T cells (Figure 3c), we observed an increase in CD8^+^ CM and a reduction in the percentage of CD8^+^ EM and CD8^+^ EM CD279/PD-1^+^ in both studied groups of patients compared to HCs. In addition, we found an exhausted landscape, with an increased percentage in CD8^+^ TEMRA CD279/PD-1^+^ (*p* = 0.003) and a simultaneous decrease in CD8^+^ naïve CD279/PD-1^+^ (*p* = 0.036) in ECMO_D compared to ECMO_S patients, while in terms of absolute number, we did not find any significant difference between survived and deceased patients, given the very low number of circulating lymphocytes in both groups. On the other hand, the immune exhaustion in ECMO_D patients is corroborated by the increased mRNA expression of activator protein-1 (AP-1) transcription factor subunits (JUNB, JUND, FOS, FOSL2, ATF4, ATF6) [16,25] compared to ECMO_S patients (Supplementary Figure S5). Analyzing CD3^+^ T cells, we found a significant increase of CD4^+^CD8^+^ T cells in ECMO_S compared to ECMO_D patients (*p* = 0.034) and a significant reduction of total γδ T cells in both ECMO_S (*p* = 0.003) and ECMO_D patients (*p* = 0.025) compared to HCs (Supplementary Figure S6a). Concerning MAIT and Th17 lymphocytes (Supplementary Figure S6b), the analysis revealed a significant reduction of the former in both groups of patients compared to HCs, while Th17 was significantly decreased in ECMO patients who later died compared to both surviving counterparts and HCs. As seen in Supplementary Figure S6c, we observed a significant reduction of Tγδ1 cytotoxic (CD57^+^) cells in both ECMO_S (*p* = 0.0007) and ECMO_D patients (*p* = 0.038) compared to HCs, while, conversely, there was an increase in Tγδ2 cytotoxic (CD57^+^), which was only significant in ECMO_S patients (*p* = 0.02). The analysis of NK cells (Supplementary Figure S6d) evidenced a significant increase in NK maturing cells from ECMO_S patients compared with both HCs (*p* = 0.003) and ECMO_D patients (*p* = 0.011). Finally, B lymphocytes (Figure S7e) showed no significant variation among the three groups of patients, except for double-negative late memory B cells, associated with an exhausted phenotype [26] and worse COVID-19 outcome [27], which were significantly increased in ECMO_D patients compared with both HC (*p* = 0.013) and ECMO_S (*p* = 0.041) groups. Supplementary Figure S7 illustrates the box plot of all statistically significant immune cell subpopulations.

### 2.5. A Machine-Learning Time-Dependent Approach Identified a Predictive Signature of Death for ECMO Patients

**Figure 4:**
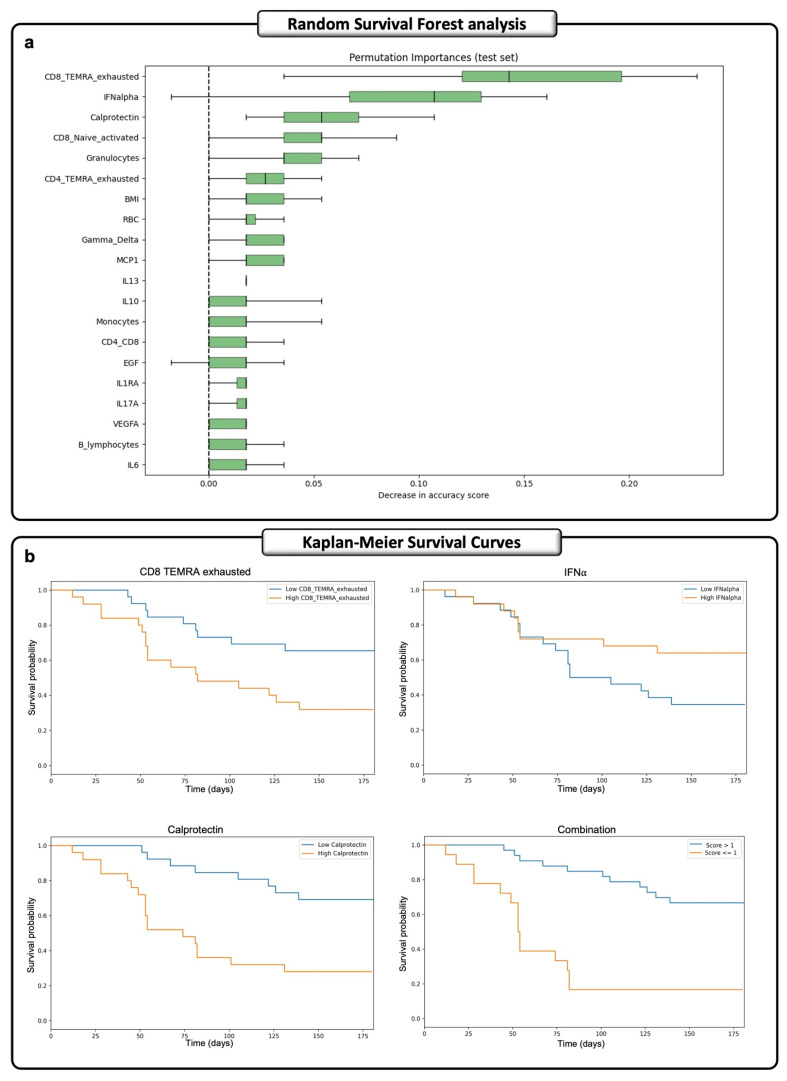
A machine learning time-dependent survival analysis of 69 different features revealed possible predictor markers associated with patient mortality during ECMO treatment. (**a**) Evaluation of the random survival forest (RSF) score to identify critical factors influencing patient survival over 180 days. The dataset includes 69 variables derived from clinical data, cytokine expression, and flow cytometry subpopulations of 51 ECMO patients (24 ECMO_S and 27 ECMO_D). Feature importance was estimated using permutation, wherein the values of each predictor variable were randomly shuffled to assess their impact on the model’s performance in predicting patient survival. (**b**) Kaplan–Meier curves of the effect of each variable (exhausted CD8 TEMRA, IFNα, and calprotectin) and of the combination of the three features together, identified by RSF, show the survival probabilities over 180 days. The dataset is divided into two groups according to the median value of each feature. ECMO_S: ECMO survived; ECMO_D: ECMO deceased.

The RSF analysis, a machine learning statistical approach, on 51 ECMO patients (24 ECMO_S, 27 ECMO_D), revealed possible predictive markers associated with patient mortality during the 180 days under ECMO treatment. Figure 4a shows the 20 most significant variables for predicting patient outcomes among the 69 examined features. These variables were identified using the permutation importance feature selection method. The concordance index (C-index) score for the model is 0.927 for the training test, the mean time-dependent area under the receiver operating curve (td-AUC) is 0.96, while the Brier score at day 180 is 0.073, with a mean value of 0.169, confirming the effectiveness of the model (Supplementary Figure S8). To create a predictive score, we focused on the three most important features identified through the RSF approach: exhausted CD8^+^ TEMRA T cells, IFNα, and calprotectin. We first evaluated their prognostic impact alone and then created a combined score, considering their impact in predicting the patient’s outcome (Figure 4b). On univariate analysis, we found high levels of exhausted CD8^+^ TEMRA T cells, low IFNα, and high calprotectin levels linked to worse survival (HR: 2.648, 95% CI = 1.177 to 5.955, *p* = 0.018; HR: 0.483, 95% CI = 0.214 to 1.086, *p* = 0.078; HR: 3.918, 95% CI = 1.691 to 9.078, *p* = 0.001). Finally, in the ECMO cohort, we established an overall score by combining these three features, which was calculated as the sum of values proportional to their respective hazard ratios: a high level of exhausted CD8^+^ TEMRA T cells contributed +1, a high level of IFNα contributed −1, and a high level of calprotectin contributed +2. To assess the effectiveness of the prediction at different time points, we examined the predictive performance at intervals of 30, 60, and 180 days post-hospitalization, performing ROC analyses (Supplementary Figure S9), obtaining an AUC of 0.862 at 30 days, and for extended durations, exhibiting AUC values of 0.798 at 60 days and 0.802 at 180 days. Thus, the patients were divided into two groups based on the median value of the overall score, which was equal to 1 (33 patients with a score ≤ 1; 18 patients with a score > 1) (Supplementary Figure S10). Notably, when combined in this manner, the three features collectively demonstrated a significant effect on the survival outcome, yielding an increased hazard ratio of 5.563 (95% CI = 2.489 to 12.431, *p* = 0.00003) (Figure 4b, Combination).

## 3. Discussion

This retrospective study analyzed 80 patients with severe COVID-19-related pneumonia admitted to the ICU at IRCCS-ISMETT Hospital. All patients required ECMO support due to unresponsive ARDS. Using a comprehensive approach that included clinical and laboratory data, cytokine levels, RNA sequencing, and immune cell profiles, we aimed to identify baseline immune-based biomarkers associated with outcomes during the 180 days post-ECMO initiation. The findings revealed significant differences between ECMO patients and control cohorts, suggesting a need for refined inclusion criteria to better identify patients with distinct disease profiles. Notably, the ECMO cohort was younger, had longer hospital stays, and showed distinct biochemical and hematological profiles, including a higher incidence of bacterial co-infections and reduced hemoglobin levels. Cytokine analysis identified a signature linked to myeloid-driven immune responses and lymphocyte suppression. Consistent with the existing literature [15], an impaired cytokine profile (including IL8, NGAL, resistin, IL2R, and calprotectin) was observed, potentially contributing to erythrocyte damage, eryptosis, and coagulopathy. The cytokine network analysis showed a progressive reduction in coordinated activation patterns in ECMO patients, with the most severe disruption observed in deceased patients, likely due to an uncontrolled cytokine storm. Whole blood RNA sequencing indicated that peripheral blood immune cells, especially neutrophils, contributed to cytokine production in both surviving and deceased ECMO patients compared to healthy controls [17,18,19,20]. This suggests a possible pathogenetic model, where elevated cytokine levels drive neutrophils into the lungs and other tissues, causing significant damage through the release of granules, increasing endothelial permeability and thrombosis risk, and promoting a hypercoagulable state [28,29]. This hypothesis is supported by the observed increase in the mRNA expression of immunosuppressive/immature-like phenotype markers (PD-L1 and CD64) [16]. Alterations in the T cell compartment were observed, including an exhausted phenotype and reduced functional T cells, including an increase in exhausted PD-1^+^ TEMRA CD8 T cells, as well as reductions in central memory CD4^+^ T cells, effector memory CD4^+^ and CD8^+^ T cells, MAIT, and Th17 cells. The presence of an exhausted T cell phenotype, as previously reported in COVID-19 [30,31], and the absence of fresh T cells capable of mounting an effective anti-viral immune response may render the time gained through ECMO oxygenation futile and counterproductive, as patients may fail to recover from the main infection and become exposed to more infectious agents without an effective response, thereby increasing the risk of death. Moreover, the presence of increased transcriptional expression of the AP-1 complex [16], especially in deceased patients, and high circulating levels of IL2R in critical COVID-19 patients further support T cell exhaustion due to hyper-reactivation leading to impaired functional cytolytic activity [32,33]. Additionally, soluble IL2R can act as a decoy receptor, reducing the bioavailability of IL-2 [34], which could further reduce the number of circulating lymphocytes. Despite no significant B cell alterations, ECMO patients exhibited increased serum IgG production (both anti-S and anti-N serum IgG) and an increase in exhausted double-negative B cells, as previously reported [26,27], which may indicate an attempt to rescue the patient from infection by unsuccessfully over-activating the B cell subpopulation. In conclusion, although our study was conducted in the pre-vaccination era and during early SARS-CoV-2 circulation, the immune signatures identified likely reflect a convergent high-risk immunopathological phenotype of severe ARDS requiring ECMO support, which may remain relevant across different viral variants and in selected vaccinated populations progressing to critical disease. Finally, attempts to build a predictive score for ECMO outcomes using machine learning identified three variables (number of PD1^+^TEMRA CD8 T cells, plasma IFNα, and calprotectin levels) that collectively could reduce the risk of death by 5.56-fold under ECMO treatment. Our goal was to create a non-invasive and easy-to-use score that could be incorporated into the clinical routine once validated in larger series. This score could help identify patients who would truly benefit from ECMO treatment, reducing unnecessary mortality and costs [35].

## 4. Materials and Methods

### 4.1. Study Cohorts and Inclusion and Exclusion Criteria

The study involved 80 patients with severe COVID-19-related pneumonia, admitted to the intensive care unit (ICU) of IRCCS-ISMETT Hospital, who required ECMO support due to unresponsive ARDS. The patients were managed between September 2020 and April 2021, before the advent of COVID-19 vaccines, and were infected with the original SARS-CoV-2 Wuhan strain. All patients received standard-of-care treatment during the study period, including systemic corticosteroids administered before or at ICU admission, while remdesivir was used selectively. Targeted immunomodulatory therapies were not routinely used and were therefore not systematically recorded. Respiratory indications for venovenous (V-V) ECMO were evaluated only after failure of recruitment maneuvers, conventional protective invasive mechanical ventilation, prone positioning, diuresis, or renal replacement therapy for correction of volume overload [1]. Acuity and reversibility of pulmonary injury were the essential conditions to proceed with V-V ECMO support when the following criteria were satisfied: optimization of mechanical ventilation; Murray score > 3; PaO_2_/FiO_2_ ratio < 100 mmHg, despite high PEEP > 10 cm H_2_O on FiO_2_ > 80%; thoracic-pulmonary compliance (CT stat) < 30 mL/cm H_2_O; severe hypercapnia with pH < 7.25, despite optimal conventional invasive mechanical ventilation (respiratory rate 35 bpm and plateau pressure ≤ 30 cm H_2_O). Patients on mechanical ventilation for longer than 7 days and older than 60 years were generally not considered for extracorporeal support. Blood samples (whole blood, serum, and plasma) were collected at baseline, defined as within 48 h from ICU admission and before or at the time of ECMO cannulation. In the majority of patients (75%), samples were obtained before ECMO initiation, during the phase of refractory hypoxemia under maximal conventional respiratory support. In the remaining patients (25%), sampling was performed on the same day as ECMO cannulation and always within 24 h of cannulation. No samples were collected beyond 24 h after ECMO initiation. We obtained demographic data, as well as information about comorbidities, from electronic health records. We excluded from the study patients with HIV, cancer, rheumatologic disorders, or autoimmunity diseases and those using monoclonal antibodies. We also included a control cohort for comparative analysis, including 23 never-infected healthy controls (HCs). As reported in Table 1, our HC cohort includes people age- and gender-matched with ECMO patients. The study was conducted in accordance with the Declaration of Helsinki and approved by the IRCCS ISMETT Institutional Research Review Board (IRRB 00/21) and the ISMETT Ethics Committee. Written informed consent was waived due to the emergency context of the COVID-19 pandemic, as critically ill patients were frequently unable to provide valid consent and extraordinary organizational constraints prevented its systematic collection. The waiver was granted in accordance with national emergency legislation (Decreto-Legge 17 March 2020, n. 18, art. 17, and subsequent emergency provisions) and Regulation (EU) 2016/679 (GDPR, Art. 9(2)(c) and 9(2)(j)), which allow the processing of health data without consent for reasons of public interest in public health and protection of vital interests. Whenever feasible, patients were informed at hospital discharge and were given the opportunity to object to the use of their data, in accordance with institutional policies.

### 4.2. Laboratory Analyses

Blood tests were performed in blood samples collected in a Vacutainer K2-EDTA tube (Becton Dickinson, San Jose, CA, USA) using an XN-2000 automated hematology analyzer (Sysmex, Norderstedt, Germany). SARS-CoV-2 anti-S and anti-N IgG were detected in serum from 42 ECMO (20 ECMO_S and 22 ECMO_D) patients and in all HCs. The LIAISON^®^ SARS-CoV-2 S1/S2 IgG (DiaSorin S.p.A., Saluggia, Italy) was used on the fully automated LIAISON^®^ XL Analyzer (DiaSorin S.p.A., Saluggia, Italy) and expressed as binding antibody units (BAU/mL); values > 33.8 BAU/mL were considered positive. Anti-nucleocapsid (N) IgG levels were measured using the semi-quantitative SARS-CoV-2 IgG chemiluminescent microparticle immunoassay (CMIA) on Architect i2000SR (Abbott, Lake Forest, IL, USA). An index value (obtained by signal sample/cut-off (S/CO) ratio) of ≥1.4 was considered positive.

### 4.3. Cytokinome Profile

COVID-19 pathogenesis and severity are dependent on a hyper-inflammatory response leading to a “cytokine storm”. Intending to understand the complex dynamics of the cytokine network (cytokinome) and its potential predictive role in the fatal outcome of SARS-CoV-2 infection, we examined a panel of 35 circulating cytokines, including chemokines, interleukins, and growth factors, in the baseline plasma samples available from 80 ECMO patients (36 ECMO_S and 44 ECMO_D) and from 23 never-infected/vaccinated HCs (Supplementary Figure S1). After normalization (Supplementary Figure S2), data were clustered to identify subgroups based on specific cytokine profiles. The concentrations of selected proteins in the plasma were measured by using magnetic bead technology from Luminex^TM^, with the ProcartaPlex Human Magnetic Luminex Kits (Affymetrix, Wien, Austria), with 2 panels containing a total of 34 analytes: Regulated on Activation Normal T Expressed and Secreted (RANTES or CCL5), resistin, hepatocyte growth factor (HGF), neutrophil gelatinase-associated lipocalin (NGAL or lipocalin-2, LCN2), interleukin (IL) 1β, IL1RA, IL2, IL2R, IL4, IL5, IL6, IL7, IL8, IL10, IL12p40, IL13, IL15, IL17A, interferon (IFN)α, IFNγ, tumor necrosis factor (TNF)α, epidermal growth factor (EGF), basic fibroblast growth factor (FGF2), granulocyte-macrophage colony-stimulating factor (GM-CSF), granulocyte colony-stimulating factor (G-CSF), matrix metallopeptidase 8 (MMP8 or neutrophil collagenase), eotaxin (CCL11), monocyte chemotactic protein-1 (MCP1 or CCL2), macrophage inflammatory protein-1 alpha (MIP1α, CCL3), MIP1β (CCL4), monokine induced by interferon-gamma (MIG or CXCL9), interferon gamma-induced protein 10 (IP10 or CXCL10), vascular endothelial growth factor A (VEGFA), and VEGFD. The assays were conducted according to the manufacturer’s instructions. Results were obtained with Luminex^TM^ 200 Systems (Luminex Corporation, Austin, TX, USA). Fluorescence intensity (FI) data from the assays were used for further analysis. Calprotectin was detected using a DiaSorin Calprotectin Chemiluminescent Immunoassay (CLIA) on a Liaison XL analyzer (DiaSorin S.p.A., Saluggia, Italy; serum/plasma protocol research use only). Imprecision analysis was done using kit iQC (low/high) and patient pool plasma (low, medium, and high), and the results were expressed as µg/mL. The statistical analysis used is reported in the Supplementary Materials and Methods, Section S1.1 [36].

### 4.4. Whole Blood Total RNA-Seq

To better characterize the dynamics of cytokine production in ECMO patients and to determine whether their excessive production is exclusively inflamed tissue-dependent (e.g., pulmonary tissue) or also from peripheral blood immune cells, we carried out whole-blood total RNA sequencing. The whole venous blood from 2 healthy controls and 6 representative ECMO patients (3 ECMO_S and 3 ECMO_D) was collected in PAXgene Blood RNA tubes (QIAGEN, Hilden, Germany) and stored at −80 °C, following the manufacturer’s instructions. Briefly, the RNA extraction involved thawing samples, lysing cells, and purifying nucleic acids. The RNA yield was estimated by measuring the absorbance at 260 nm in a NanoDrop 2000 spectrophotometer (Life Technologies, Carlsbad, CA, USA). RNA purity was calculated from the ratio of absorbance at 260 and 280 nm, while RNA integrity was assessed using the 4200 Tape Station System (Agilent Technologies, Palo Alto, CA, USA), which provides RNA integrity number (RIN) scores for RNA quality control. Finally, the libraries were generated for sequencing using a TruSeq mRNA V2 sample preparation kit with Ribo-Zero Gold (Illumina, San Diego, CA, USA), following the manufacturer’s instructions. A Qubit 2.0 Fluorometer (Life Technologies, Carlsbad, CA, USA) and 4200 Tape Station System (Agilent Technologies, Santa Clara, CA, USA) were used to assess the quality and yield of the prepared libraries. Sequencing was done on a NextSeq™ 550 system (Illumina, San Diego, CA, USA) with 2 × 76 cycles, following the manufacturer’s instructions. Quality control measures included FastQC (v0.11.9, Babraham Institute) and Trimmomatic (v0.32) for preprocessing. The raw data were mapped to the human reference genome hg19 using STAR (v2.7.0). Transcript abundances were estimated using RSEM (v1.3.3), and gene expression levels were normalized by computing transcripts per kilobase million (TPM). A z-score was computed to compare the circulating levels and mRNA expression of cytokines in whole blood cells.

### 4.5. CIBERSORTx Analysis

The CIBERSORTx tool [37] was utilized to estimate the cellular composition of each patient based on whole blood RNA sequencing data. A reference matrix was constructed using the expression profiles from Xie et al. [38], which encompassed B-lymphocytes, natural killer (NK) cells, CD8^+^ and CD4^+^ T-lymphocytes, neutrophils, and monocytes. Subsequently, the cellular composition of 2 HCs, 3 ECMO_S patients, and 3 ECMO_D patients was estimated and presented through bar plots. Additionally, a correlation heatmap was generated to examine the interrelationship between different cellular types.

### 4.6. Flow Cytometry

For flow cytometry, whole blood aliquots, from a Vacutainer K2-EDTA tube (Becton Dickinson, San Jose, CA, USA), of 14 HCs, 26 ECMO_S patients, and 28 ECMO_D patients were stained with 5 different combinations of anti-human fluorescent monoclonal antibodies in Brilliant Stain Buffer (BD Biosciences, Franklin Lakes, NJ, USA) (Supplementary Table S1). For each patient, we prepared the following tubes: T cell tube, MAIT/Th17 tube, Tγδ tube, B cell tube, and NK/NKT tube. Pharm Lyse solution (Becton Dickinson, San Jose, CA, USA) was used to remove red blood cells. The cells were then washed, and at least 10^5^ cells were acquired using a 16-color FACS Celesta SORP flow cytometer (Becton Dickinson, San Jose, CA, USA) with the same instrument setting. The gating strategies and the subpopulation analysis are shown in the Supplementary Materials and Methods, Section S1.2 [23,39,40].

### 4.7. Random Survival Forest (RSF) Analysis

Finally, we aimed to identify the critical factors influencing patient survival through a general dataset comprising 69 features derived from the clinical data, cytokine expression, and flow cytometry results for 51 ECMO patients (24 ECMO_S, 27 ECMO_D). To address non-linear relationships and interactions among variables, we utilized random survival forest (RSF) [41] analysis to build a predictive model for patient survival over 180 days, taking into account patients’ outcomes along with their event date (death). To this end, scikit-survival (version 0.21.0) and scikit-learn (version 1.2.2) packages in Python (version 3.8.10) were employed. The dataset was divided into training (75%) and validation (25%) sets for model construction and assessment, respectively. Feature importance was estimated using permutation, wherein the values of each predictor variable were randomly shuffled to assess their impact on the model’s performance in predicting patient survival. The model’s goodness was evaluated by calculating the concordance index (C-index), the time-dependent area under the receiver operating curve (td-AUC) on the test set, and the Brier score over 180 days from the time of hospitalization. We then performed survival analysis using the Kaplan–Meier technique with the lifelines library (version 0.27.7), to assess individual features and combined effects in survival outcomes. For single-feature analysis, Kaplan–Meier curves were generated for the three most important features, dividing the dataset into lower and higher values than the median. To determine statistical significance, we conducted the pairwise log-rank test for each curve. Significance was considered for *p*-values less than 0.05. Furthermore, we employed Cox proportional hazard regression to assess the hazard ratio (HR) and confidence interval (CI) for each analysis. Additionally, we investigated the combined effect of the three features by creating an overall score, calculated as the sum of values proportional to the HRs of each feature. Subsequently, Kaplan–Meier survival curves were generated and visualized for both the combined analyses, providing a comprehensive view of the survival probabilities over time. Similarly, the HR for the combined analysis was computed, and statistical significance was assessed.

### 4.8. Statistical Analysis

Microsoft Office Excel (version 2305) was used for data collection. Statistical analysis were performed with Python (version 3.8.10) or R (version 4.2.2), using the packages Pandas (version 1.5.1), Numpy (version 1.23.2), UMAP (version 0.5.3), Sklearn (version 1.2.2), Seaborn (version 0.12.1), Matplotlib (version 3.5.3), combat (version 0.3.3), ggplot2 (version 3.4.2), readxl (version 1.4.2), ggpubr (version 0.6.0), ggsignif (version 0.6.4), pheatmap (version 1.0.12), corrplot (version 0.92), corrr (version 0.4.4), RColorBrewer (version 1.1.3), scales (version 1.2.1), FlowCT (version 1.0.0), Seurat (version 4.3.0), Sksurv (version 0.21.0), and lifelines (version 0.27.7), and missing values were omitted. R code and Python code are provided in the Supplementary Information. Moreover, Graph Pad Prism (version 9.0) software was used to carry out other statistical analyses. Depending on the type of samples being compared, the Wilcox test, the Mann–Whitney test, or the Kruskal–Wallis test with Dunn’s multiple comparisons was used (*p* < 0.05 was considered significant).

## 5. Conclusions

In conclusion, by employing a comprehensive approach that included cytokine profiling, RNA sequencing, and immune cell analysis, we identified key factors that influence mortality risk in ECMO patients. Notably, T cell exhaustion, reduced interferon-alpha levels, and elevated calprotectin emerged as critical predictors of poor outcomes, underscoring the pivotal role of immune dysfunction in disease progression. The findings highlight the potential of using immune profiling to guide ECMO eligibility and optimize patient selection, leading to better clinical outcomes. Moreover, the development of a predictive score incorporating these biomarkers offers a promising non-invasive tool for future clinical applications [1,7,13,42]. However, this study is limited by its retrospective design, relatively small sample size, and monocentric nature, which may affect the generalizability of the results. Future research with larger, multicentric cohorts and longitudinal analyses is necessary to validate these findings and deepen our understanding of the dynamic immune changes in ECMO patients. Despite these limitations, our study provides valuable insights into the role of immune factors in ECMO outcomes and lays the groundwork for more targeted and effective approaches in the management of critically ill COVID-19 patients.

## Figures and Tables

**Table 1:** Patient characteristics.

	ECMO Patients
Patients, n	80
Mean age ± SD, years	50.2 ± 8.8
Male (n; %)	61 (76.2%)
Hypertension (HTN) (n; %)	38 (46.2%)
Chronic Cardiac Disease (n; %)	3 (3.7%)
Diabetes (n; %)	19 (22.5%)
Obesity (n; %)	45 (56.2%)
Chronic Pulmonary disease (n; %)	9 (10%)
Chronic Kidney Disease (n; %)	3 (3.7%)
Transplantation (n; %)	1 (1.2%)
Other comorbidities (n; %)	25 (31.2%)
SOFA score (mean value ± SD)	7.9 ± 2.6
APACHE II score (mean value ± SD)	16.3 ± 6
SAPS II score (mean value ± SD)	44 ± 13
ICU-LOS, days (mean value ± SD)	71.8 ± 56
Death (n; %)	44 (55%)

## Data Availability

The raw data supporting the conclusions of this article will be made available by the authors, without undue reservation, to any qualified researcher. The raw RNA-seq datasets generated and/or analyzed during the current study are available in the NCBI repository, accession code PRJNA1354675.

## Supplementary Materials

The following supporting information can be downloaded at: https://www.mdpi.com/article/10.3390/ijms27010390/s1.
