# Supplementary material for: Immune-Based Biomarkers as Predictors of Mortality in ECMO Therapy for Severe COVID-19 ARDS: Insights from a Retrospective Study
Source: Int J Mol Sci. 2025 Dec 30;27(1):390. doi: 10.3390/ijms27010390 (PMC12786001; doi:10.3390/ijms27010390)
Supplement: Supplementary file 1 [file ijms-27-00390-s001.zip › ijms-4012093-supplementary.pdf]

Supplementary Material

Article

Immune-Based Biomarkers as Predictors of Mortality in ECMO Therapy for Severe COVID-19 ARDS: Insights from a Retrospective Study

Rosalia Busà [1,*,†], Giovanna Panarello [1,†], Alessia Gallo [1], Vitale Miceli [1], Salvatore Castelbuono [1,2], Maria Concetta Sorrentino [1], Giandomenico Amico [3], Claudia Carcione [3], Giovanna Russelli [1], Nicola Cuscino [1], Monica Miele [3], Francesca Timoneri [3], Mariangela Di Bella [3], Giovanni Zito [1], Floriana Barbera [1], Ester Badami [3], Anna Maria Corsale [4], Mojtaba Shekarkar Azgomi [5], Pier Giulio Conaldi [1], Cirino Botta [4] and Matteo Bulati [1,*]

1 IRCCS ISMETT, 90127 Palermo, Italy; gpanarello@ismett.edu (G.P.); agallo@ismett.edu (A.G.); vmiceli@ismett.edu (V.M.); scastelbuono@ismett.edu (S.C.); msorrentino@ismett.edu (M.C.S.); grusselli@ismett.edu (G.R.); ncuscino@ismett.edu (N.C.); gzito@ismett.edu (G.Z.); fbarbera@ismett.edu (F.B.); pgconaldi@ismett.edu (P.G.C.)
2 Department of Engineering, University of Palermo, 90128 Palermo, Italy
3 Ri.MED Foundation, 90133 Palermo, Italy; gamico@fondazionerimed.com (G.A.); ccarcione@fondazionerimed.com (C.C.); mmiele@fondazionerimed.com (M.M.); ftimoneri@fondazionerimed.com (F.T.); mdibella@fondazionerimed.com (M.D.B.); ebadami@fondazionerimed.com (E.B.)
4 Department of Health Promotion, Mother and Child Care, Internal Medicine and Medical Specialties, University of Palermo, 90127 Palermo, Italy; annamaria.corsale@unipa.it (A.M.C.); cirino.botta@gmail.com (C.B.)
5 Department of Biomedicine, Neuroscience and Advanced Diagnostic Diagnostic (BIND), University of Palermo, 90133 Palermo, Italy; mojtaba.shekarkarazgomi@unipa.it
* Correspondence: rbusa@ismett.edu (R.B.); mbulati@ismett.edu (M.B.)
† These authors contributed equally to this work.

S1. Supplementary Materials and Methods

S1.1. Cytokinome statistical analysis

A cytokinome dataset comprising the expression levels of 35 cytokines from 103 patients (23 HC, 36 ECMO_S, and 44 ECMO_D) was constructed. To address batch effects in the cytokinome data, the pyComBat package (version 0.3.3) for Python (version 3.8.10) was employed for correction. Subsequently, the original and corrected datasets were subjected to Uniform Manifold Approximation and Projection (UMAP) visualization using the UMAP package (version 0.5.3) for Python. Next, the batch-corrected cytokine expressions were scaled to a range between 0 and 1. Thus, to visualize the patterns of cytokine expression and their interactions, a clustered heatmap was generated using the heatmap package (version 1.0.12) for R (version 4.2.2); patient gender and condition were annotated in the heatmap, and reported in the image legend. Afterward, a pairwise Pearson correlation analysis was conducted between cytokines and clinical features, and the results were depicted using a correlation plot with the corr-plot package (version 0.92). Network plots for each condition were also created using the ggplot2 package (version 3.4.2). Statistically significant correlations were determined based on the 95% confidence interval (CI). Furthermore, cytokine levels for each condition were assessed by generating boxplots with the ggplot2 package, along with a trend line, and the pairwise statistical significance of condition differences was evaluated using the Wilcox test. Significance was considered at a p-value < 0.05, and only significant differences were depicted.

S1.2 Flow cytometry statistical analysis

The flow cytometry data were analyzed to undertake a clustering analysis, aiming to identify different immune cell populations. First, the Infinicyt software (version 2.0.6; Cytognos SL, Salamanca, Spain) was utilized to detect and eliminate undesirable debris and doublet cells based on the FSC-A/FSC-H dot plot. The data were then analyzed using the R package FlowCT

(version 1.0.0). A random selection of 50000 events was made from each subject for T and NK/NKT tubes, while 80000 events were extracted for B, MAIT/Th17, and Tγδ tubes. Subsequently, batch effects were removed through the application of the Harmony method using the FlowCT batch correction function. The processed data were subjected to UMAP dimensional reduction and clustered using the Self-Organizing Map (SOM) method. The clustered data were exported for further evaluation in the FlowJo software (version 10). Unwanted cell populations were removed, and specific labels were manually assigned to the remaining clusters, defining major cell populations. A second batch-effect removal step was performed using Seurat (version 3) to conduct sub-clustering analysis, aiming to identify subpopulations for each cell type. From the T cell tube, based on physical parameters and markers profile, we preliminarily identified 6 major subpopulation groups: $CD3^+CD4^+$ T, $CD3^+CD8^+$ T, HLA-DR^+ (B cells, in the region of lymphocytes, and monocytes), granulocytes through the physical parameters, and erythrocytes/debris. From the same tube, based on CD3 positivity, we identified 5 major subpopulations: T $CD4^+$, T $CD8^+$, T $CD4^+CD8^+$, NKT ($CD4^-CD8^-CD57^+$), and Tγδ ($CD4^-CD8^-CD57^+$) cells. To further investigate the specific T cell subpopulations, we additionally clustered $CD4^+$ and $CD8^+$ T lymphocytes separately. For $CD4^+$ T cells, we identified 6 sub-clusters: CM (central memory, $CD45RA^-CD62L^+CD27^+CD28^+$), CM act (central memory activated, $CD38^+$), EM (effector memory, $CD45RA^-CD62L^-CD27^+CD28^+$), EM act (effector memory activated, $CD38^+$), CD4 naïve ($CD45RA^+CD62L^+CD27^+$CD28+), and TEMRA/exhausted (terminally differentiated, $CD45RA^+CD62L^-CD27^-CD28^-CD57^+$CD279/PD-$1^+$). While for $CD8^+$ T cells, we identified 7 subclusters, including CM ($CD45RA^-CD62L^+CD27^+CD28^+$), EM ($CD45RA^-CD62L^-CD27^+CD28^+$), EM CD279/PD-$1^+$, naïve ($CD45RA^+CD62L^+CD27^+CD28^+$), naïve CD279/PD-$1^+$, TEMRA ($CD45RA^+CD62L^-CD27^-CD28^-$), and TEMRA/exhausted ($CD57^+$CD279/PD-$1^+$). From the MAIT/Th17 tube we identified MAIT ($CD3^+CD8^+CD161^+$ $CD196^+TCRV\alpha7.2^+$) and Th17 ($CD3^+CD4^+CD161^+CD196^+$) lymphocytes. Regarding the Tγδ tube, we obtained the following 6 subclusters: Tγδ1 ($CD3^{low}TCRV\delta1^+$CD279/PD-1^+), cytotoxic Tγδ1 ($CD3^+TCRV\delta1^+$CD279/PD-1^+CD57^{high}CD159a/$NKG2A^{high}$), exhausted Tγδ1 ($CD3^+TCRV\delta1^+$CD279/PD-

1^{+}CD57^{+}CD159a/NKG2A^{+}), Tγδ2 (CD3^{+}TCRVδ2^{+}), cytotoxic Tγδ2 (CD3^{+}TCRVδ2^{+}CD57^{+}CD159a/NKG2A^{+}), and exhausted Tγδ2 (CD3^{+}TCRVδ2^{+}CD279/PD-1^{high}). The B cell tube allowed us to identify 8 different subpopulations: naïve (CD19^{+}IgD^{+}CD27^{-}CD69^{-}), activated naïve (CD19^{+}IgD^{+}CD27^{-}CD69^{+}), unswitched memory (CD19^{+}IgD^{+}CD27^{+}CD69^{-}), unswitched activated memory (CD19^{+}IgD^{+}CD27^{+}CD69^{+}), switched memory (CD19^{+}IgD^{-}CD27^{+}CD69^{-}), activated switched memory (CD19^{+}IgD^{-}CD27^{+}CD69^{+}), late memory/double negative (DN, CD19^{+}IgD^{-}CD27^{-}CD69^{-}), and plasmablasts (CD19^{low}CD27^{high}CD38^{high}CD69^{+}). The NK/NKT tube revealed the following 8 subpopulations: immature NK (CD3^{-}CD16^{-}CD56^{+}NKG2A^{+}CD137^{+}), maturing NK (CD3^{-}CD16^{+}CD56^{+}NKG2A^{+}NKG2D^{+}TRAIL^{+}), maturing NK CD57^{+} (CD3^{-}CD16^{+}CD56^{+}NKG2A^{+}NKG2D^{+}TRAIL^{+}CD57^{+}), mature NK (CD3^{-}CD16^{high}CD56^{+} NKG2D^{+}TRAIL^{+}), mature activated NK (CD3^{low}CD16^{low}CD56^{low}NKG2D^{+}CD57^{+}CD137^{+}), terminal NK (CD3^{-}CD16^{high}CD56^{high}NKG2D^{+}TRAIL^{+}CD57^{+}), NKT (CD3^{+}CD16^{low}CD56^{low}NKG2A^{+}TRAIL^{+}CD137^{+}), and activated NKT (CD3^{+}CD16^{low}CD56^{+}CD57^{+}CD137^{+}). Finally, the results were visualized using boxplots created with the ggplot2 R package (version 3.4.2). The Wilcox test assessed statistically significant differences between conditions, with significance at a p-value of < 0.05.

Supplementary Table S1. List of anti-human monoclonal antibodies used for flow cytometry

TUBE	Marker	Conjugation	Clone	Host/Isotype	Dilution	Manufacturer
T cells	CD3	BV711	UCHT-1	Mouse BALB/c IgG1, κ	1:20	BD Bioscience
	CD4	BV786	RPA-T4	Mouse IgG1, κ	1:20	BD Bioscience
	CD8	APC-H7	SK1	Mouse BALB/c IgG1, κ	1:20	BD Bioscience
	CD45RA	BV480	HI100	Mouse IgG2b, κ	1:20	BD Bioscience
	CD62L	BV650	DREG-56	Mouse IgG1, κ	1:20	BD Bioscience
	CD27	BV605	L128	Mouse BALB/c IgG1	1:20	BD Bioscience
	CD28	APC-R700	CD28.2	Mouse C3H x BALB/c IgG1, κ	1:20	BD Bioscience
	CD57	FITC	NK-1	Mouse IgM, κ	1:20	BD Bioscience
	CD279 (PD-1)	APC	PD1.3 1.3	Mouse IgG2bκ	1:20	Miltenyi Biotec
	HLA-DR	PE	G46-6	Mouse IgG2a, κ	1:20	BD Bioscience
	CD38	PE-CF594	HIT2	Mouse IgG1, κ	1:20	BD Bioscience
MAIT/Th17	CD3	APC-H7	SK7	Mouse BALB/c IgG1, κ	1:20	BD Bioscience
	CD4	BV786	RPA-T4	Mouse IgG1, κ	1:20	BD Bioscience
	CD8	PerCP-Cy5.5	SK1	Mouse BALB/c IgG1, κ	1:20	BD Bioscience
	CD279 (PD-1)	Vio Bright FITC	PD1.3 1.3	Mouse IgG2bκ	1:50	Miltenyi Biotec
	TCRVα7.2	PE-VIO770	REA179	Recombinant human IgG1	1:50	Miltenyi Biotec
	CD161	PE	HP-3G10	Mouse BALB/c IgG1, κ	1:20	BD Bioscience
	CD196 (CCR6)	APC	11A9	Mouse IgG1, κ	1:20	BD Bioscience
Tγδ Cells	CD3	BV711	UCHT-1	Mouse BALB/c IgG1, κ	1:20	BD Bioscience
	TCRVδ1	APC-VIO770	REA173	Recombinant human IgG1	1:50	Miltenyi Biotec
	TCRVδ2	PE-VIO770	REA771	Recombinant human IgG1	1:50	Miltenyi Biotec
	CD57	FITC	NK-1	Mouse IgM, κ	1:20	BD Bioscience
	CD159a (NKG2A)	BV605	131411	Mouse IgG2a, κ	1:20	BD Bioscience
	CD279 (PD-1)	APC	PD1.3 1.3	Mouse IgG2bκ	1:20	Miltenyi Biotec
B Cells	CD19	BV711	SJ25C1	Mouse BALB/c IgG1, κ	1:20	BD Bioscience
	IgD	PE-CF594	IA6-2	Mouse BALB/c IgG2a, κ	1:20	BD Bioscience
	CD27	BV605	L128	Mouse BALB/c IgG1	1:20	BD Bioscience
	CD69	PE-VIO770	REA824	Recombinant human IgG1	1:20	Miltenyi Biotec
	CD38	APC	HIT2	Mouse IgG1, κ	1:20	BD Bioscience
NK/NKT Cells	CD3	BV711	UCHT-1	Mouse BALB/c IgG1, κ	1:20	BD Bioscience
	CD56	BV421	NCAM16.2	Mouse BALB/c IgG2b, κ	1:20	BD Bioscience
	CD16	PE-CF594	3G8	Mouse BALB/c x DBA/2	1:20	BD Bioscience
	CD314 (NKG2D)	PerCP-eFluor700	1D11	Mouse / IgG1, kappa	1:20	Invitrogen
	TRAIL	PE	RIK-2	Mouse / IgG1, kappa	1:20	Invitrogen
	CD137	APC	REA765	Recombinant human IgG1	1:20	Miltenyi Biotec
	CD159a (NKG2A)	PE-VIO770	REA1106	Recombinant human IgG1	1:20	Miltenyi Biotec
	CD57	FITC	NK-1	Mouse IgM, κ	1:20	BD Bioscience

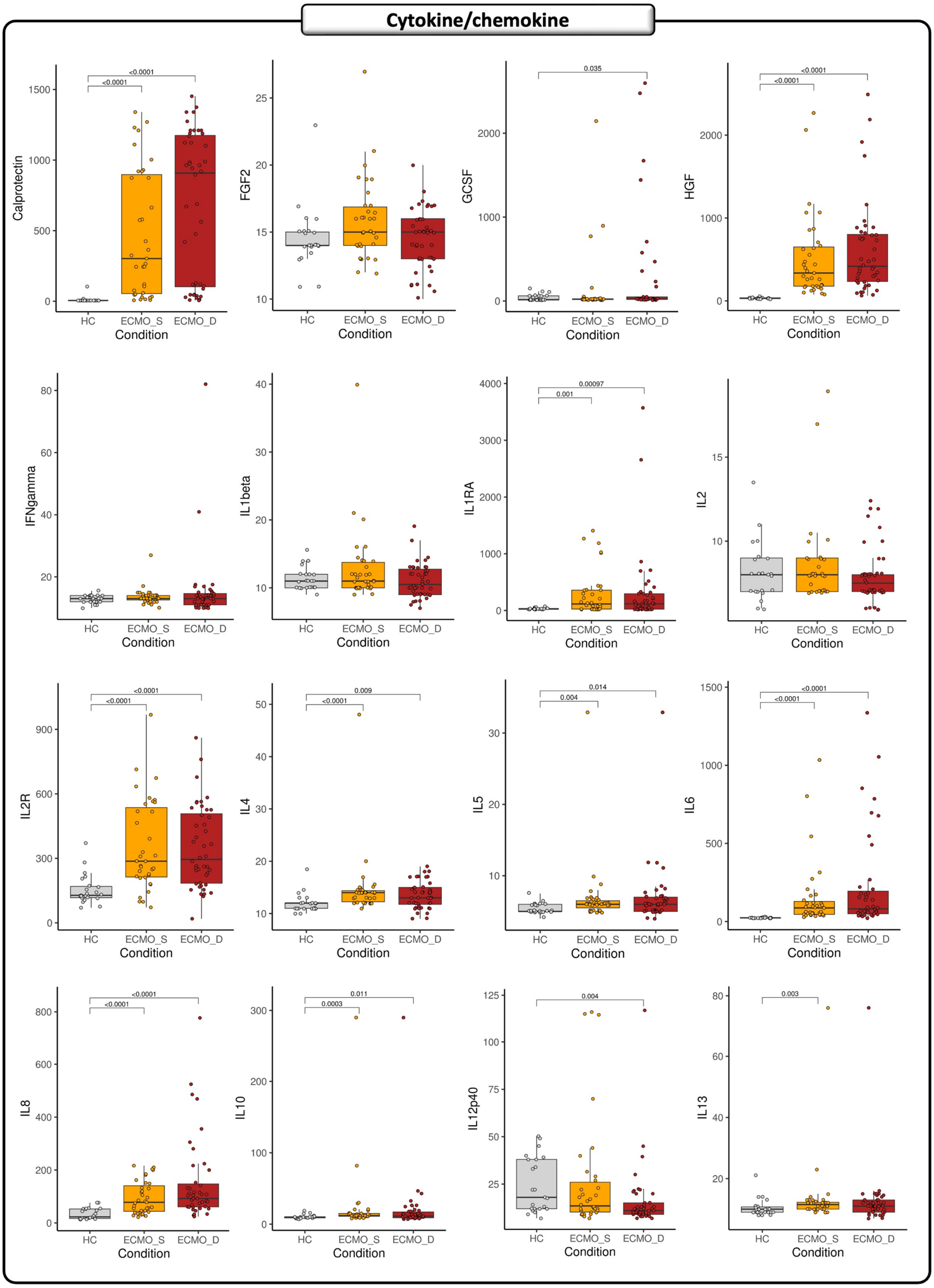
Cytokine/chemokine
Calprotectin
<0.0001
<0.0001
FGF2
GCSF
0.035
HGF
<0.0001
<0.0001
IFNgamma
IL1beta
IL1RA
0.00097
0.001
IL2
IL2R
<0.0001
<0.0001
IL4
0.009
<0.0001
IL5
0.014
0.004
IL6
<0.0001
<0.0001
IL8
<0.0001
<0.0001
IL10
0.011
0.0003
IL12p40
0.004
IL13
0.003
HC
ECMO_S
ECMO_D
Condition

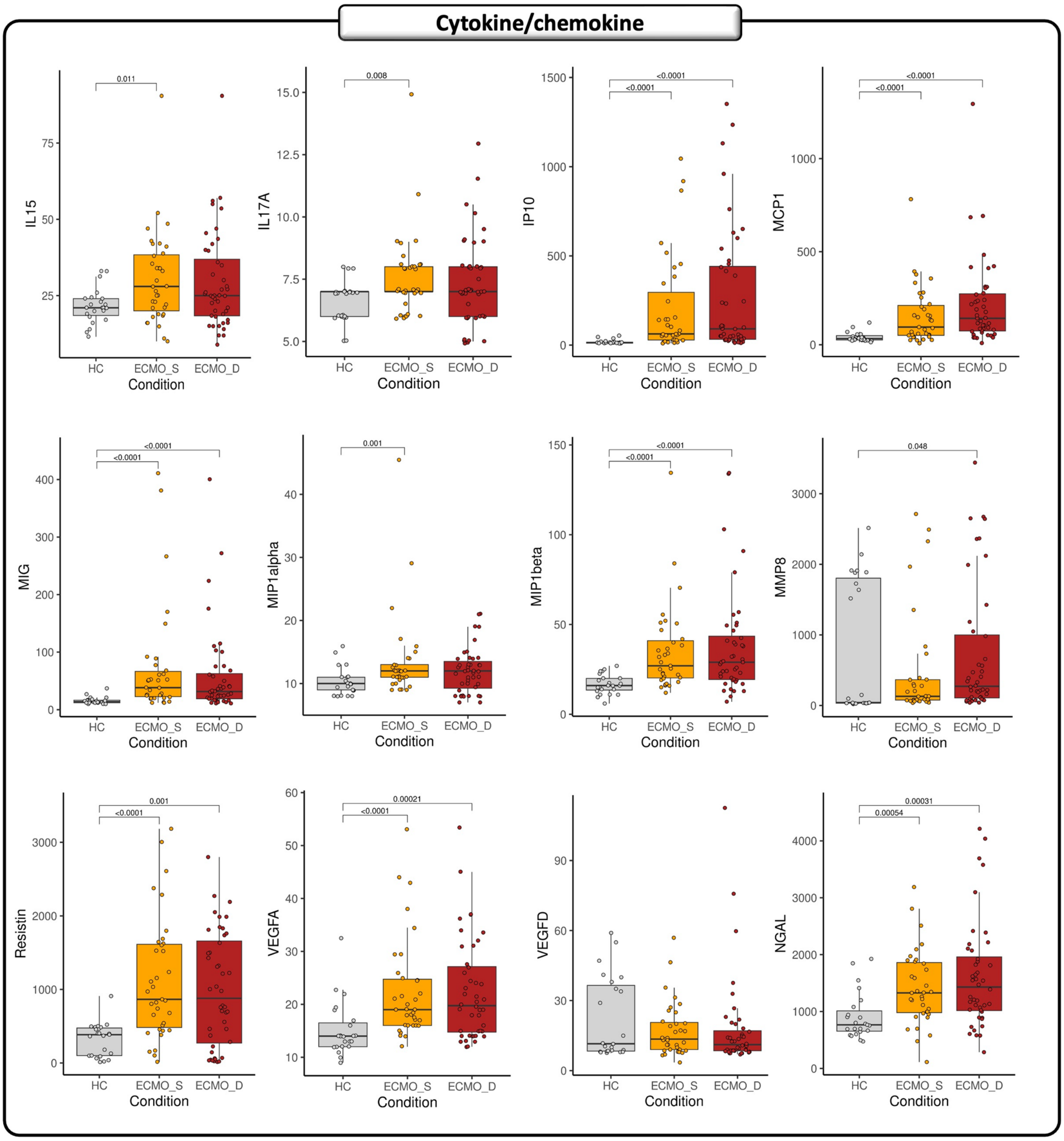

Supplementary Figure S1. **Plasma levels of selected cytokines and chemokines.** Boxplots representing the median, first, and third quartiles of plasma levels of selected cytokines and chemokines in 80 ECMO (36 ECMO_S and 44 ECMO_D) patients and 23 HC. Kruskal-Wallis test with Dunn's multiple comparisons. HC: healthy control; ECMO_S: ECMO survived patients; ECMO_D: ECMO deceased patients.

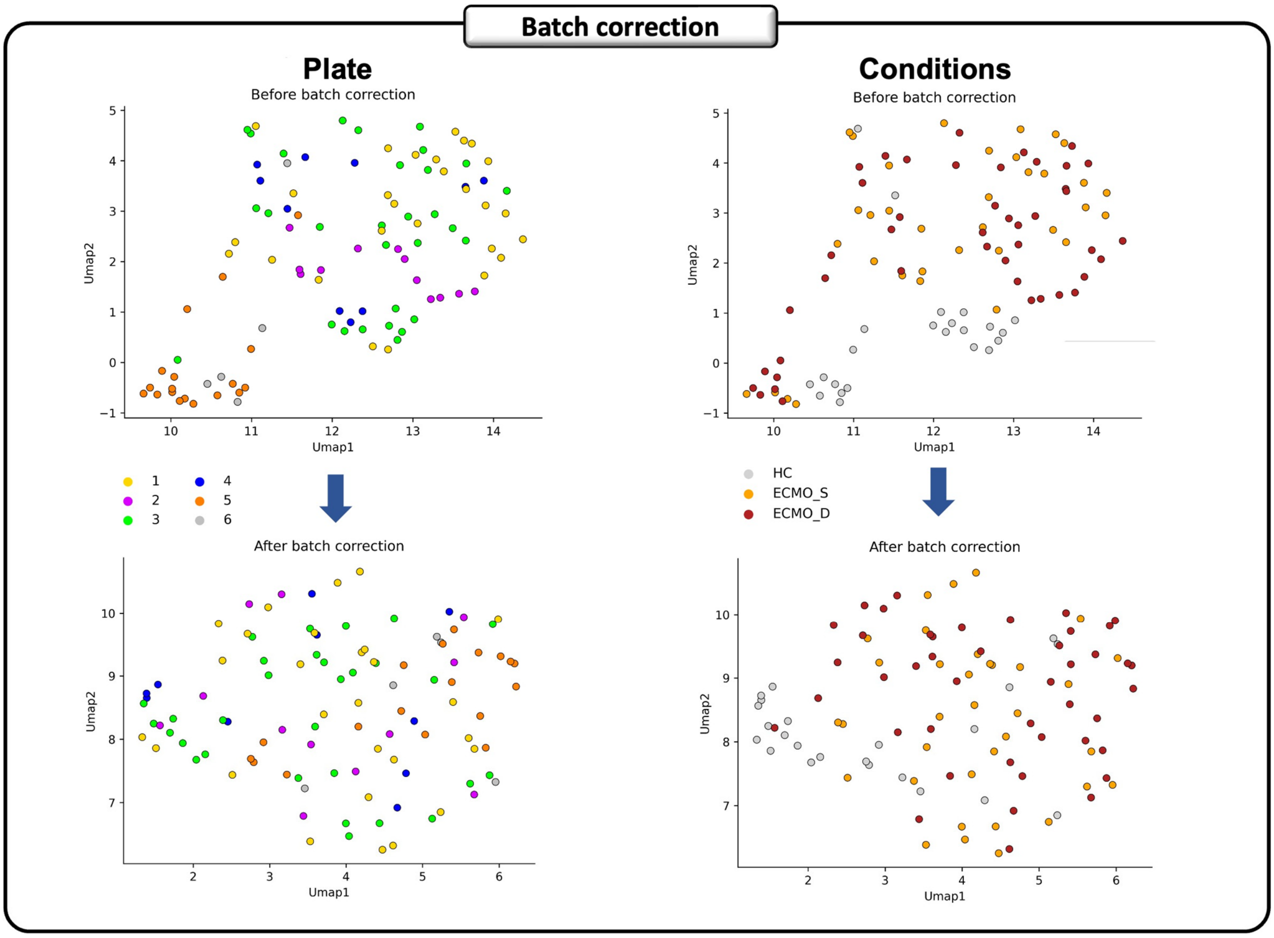

Supplementary Figure S2. **Two-dimensional UMAP correction of the cytokinome data.** Two-dimensional UMAP reduction of the cytokinome data before (first column) and after (second column) applying batch correction. Each data point represents the first and second UMAP reduction component of the 35 cytokine levels for an individual patient. (**First row**) Patients are represented by distinct colors corresponding to the plate number (1 to 6). A distinctive batch corresponding to plates five (orange) and six (gray) is present before correction. (**Second row**) Patients are categorized by condition (HC, ECMO_S, and ECMO_D). The UMAP reduction after the correction illustrates the successful mitigation of batch effects, leading to the emergence of characteristic clusters corresponding to different patient conditions (HC, ECMO_S, ECMO_D, and Mixed). HC: healthy control; ECMO_S: ECMO survived patients; ECMO_D: ECMO deceased patients.

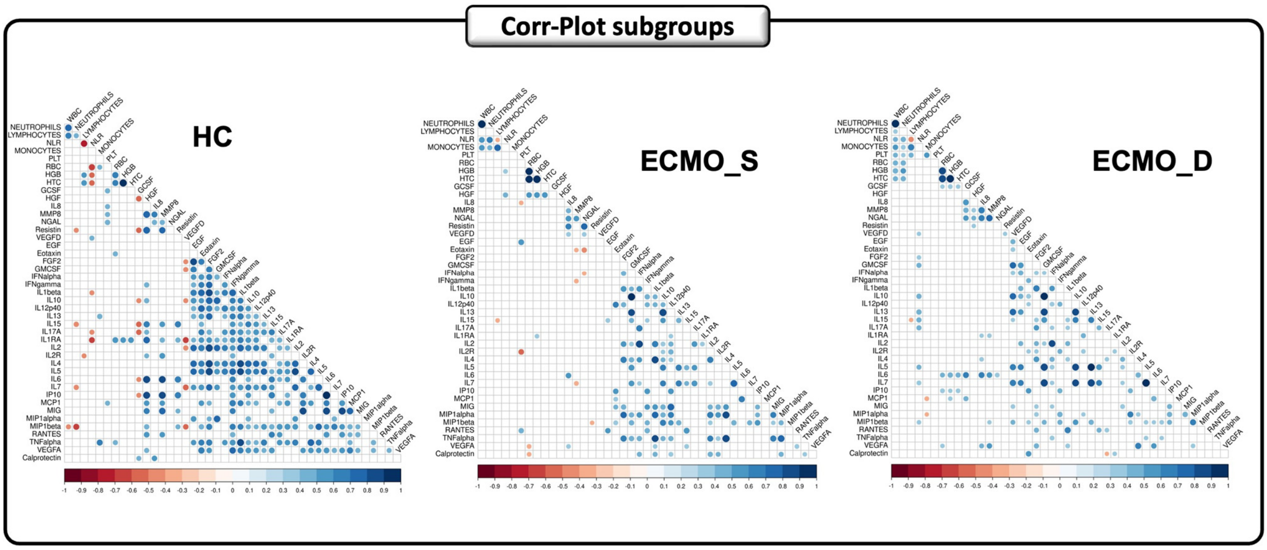

Supplementary Figure S3. Pairwise Pearson correlation matrix. Pairwise Pearson correlation matrix among all 35 circulation cytokines and 9 different clinical features, for all studied groups. Corr-plots show positive (blue) and negative (red) correlations. For a better overview, only significant differences ($p<0.05$) are displayed. F: female; M: male; HC: healthy control; ECMO_S: ECMO survived; ECMO_D: ECMO deceased.

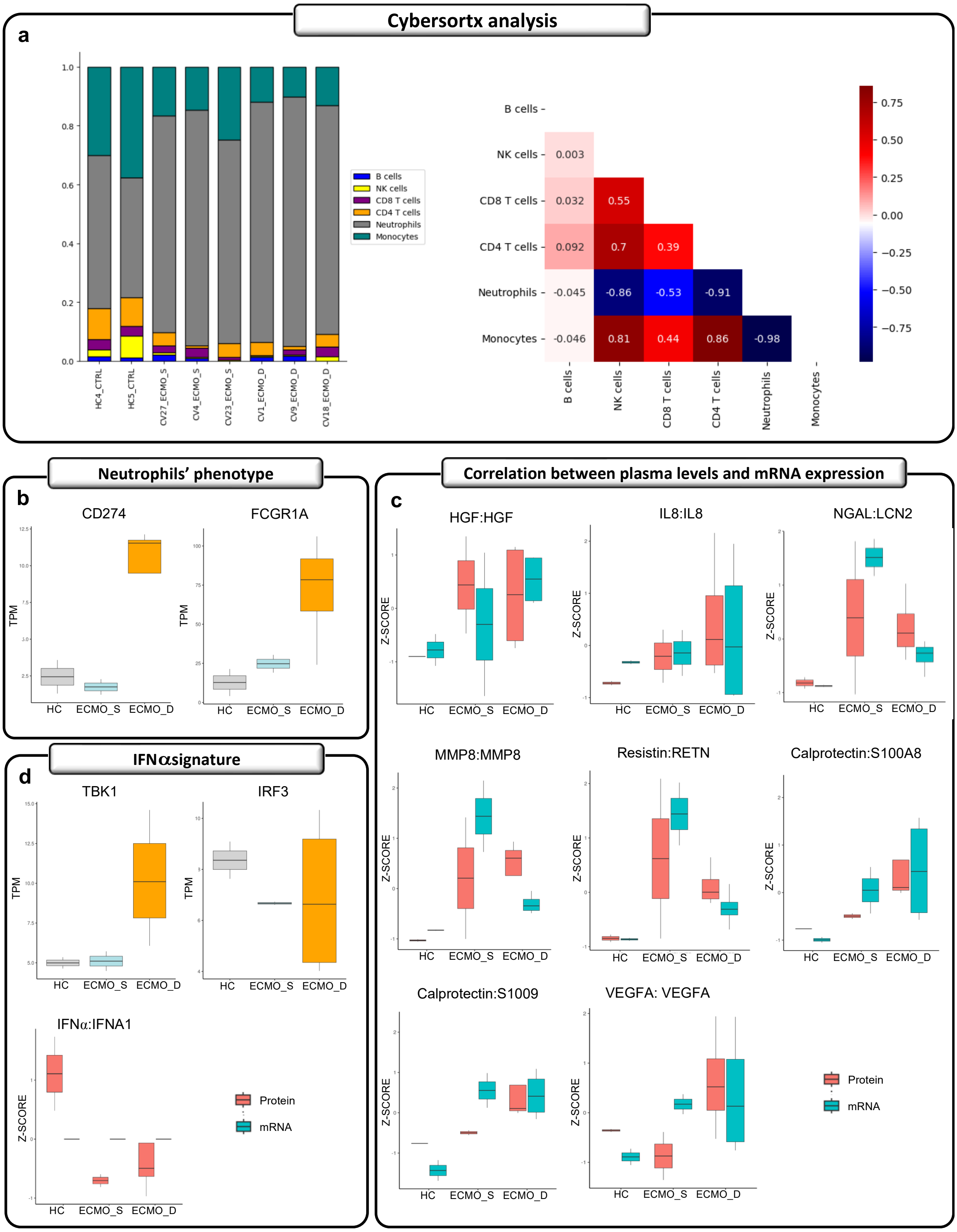

a
Cybersortx analysis
B cells
NK cells
CD8 T cells
CD4 T cells
Neutrophils
Monocytes
HC4_CTRL
HC5_CTRL
CV27_ECMO_S
CV4_ECMO_S
CV23_ECMO_S
CV1_ECMO_D
CV9_ECMO_D
CV18_ECMO_D
b
Neutrophils' phenotype
CD274
FCGR1A
TPM
HC
ECMO_S
ECMO_D
c
Correlation between plasma levels and mRNA expression
HGF:HGF
IL8:IL8
NGAL:LCN2
MMP8:MMP8
Resistin:RETN
Calprotectin:S100A8
Calprotectin:S1009
VEGFA: VEGFA
Z-SCORE
Protein
mRNA
d
IFNα signature
TBK1
IRF3
IFNα:IFNA1

Supplementary Figure S4. Whole-blood total RNA sequencing permits to discriminate of which cytokines are produced directly from peripheral blood cells or in inflamed tissue sites. **(a)** The CIBERSORTx algorithm analysis shows the cellular composition of 2 HC, 3 ECMO_S, and 3 ECMO_D patients, based on the whole blood RNA sequencing data. The bar plot (left) shows the percentage of 6 types of immune cells (B cells, NK cells, CD8 T cells, CD4 T cells, neutrophils, and monocytes) in each sample by estimating relative subsets of RNA transcripts. Pairwise Pearson correlation matrix (right panel) of the immune cells. In the heatmap, red indicates a positive correlation, and blue indicates a negative correlation. Darker colors are associated with stronger correlation coefficients. **(b)** mRNA expressions of CD274 (PD-L1) and FCGR1A (CD64), two genes characteristic of neutrophils' immature-like phenotype. The gene expression levels were normalized and expressed as median, first, and third quartiles of transcripts per kilobase million (TPM). **(c)** Comparison of circulating levels and mRNA expression of whole blood cytokines. Boxplots show the median, first, and third quartiles of z-score normalization values of each cytokine plasma level and its relative mRNA expression. **(d)** The plots are representative of IFN signature in the ECMO patients, showing the levels of the main transcription factors genes (TBK1 and IRF3), expressed as median, first, and third quartiles of transcripts per kilobase million (TPM), and the correlation between the IFN plasma levels, and IFNA1 gene expression, expressed as median, first, and third quartiles of z-score normalization values. HC: healthy control; ECMO_S: ECMO survived; ECMO_D: ECMO deceased.

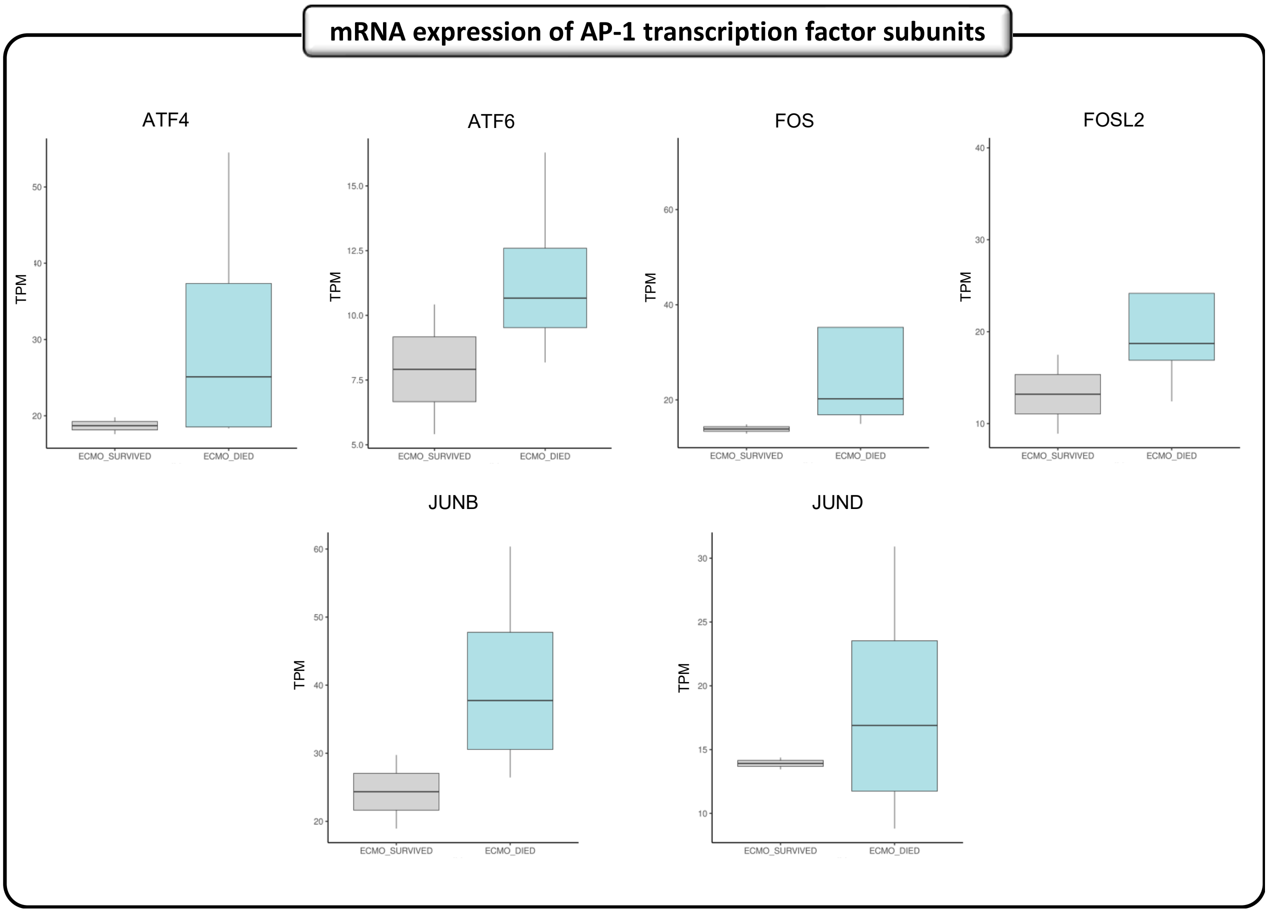

Supplementary Figure S5. mRNA expression of AP-1 transcription factor subunits. The plots show the mRNA expression of AP-1 transcription factor subunits (ATF4, ATF6, FOS, FOSL2, JUNB, and JUND), expressed as median, first, and third quartiles of transcripts per kilobase million (TPM), obtained from the whole blood RNA sequencing of 3 ECMO_S, and 3 ECMO_D subjects. ECMO_S: ECMO survived; ECMO_D: ECMO deceased.

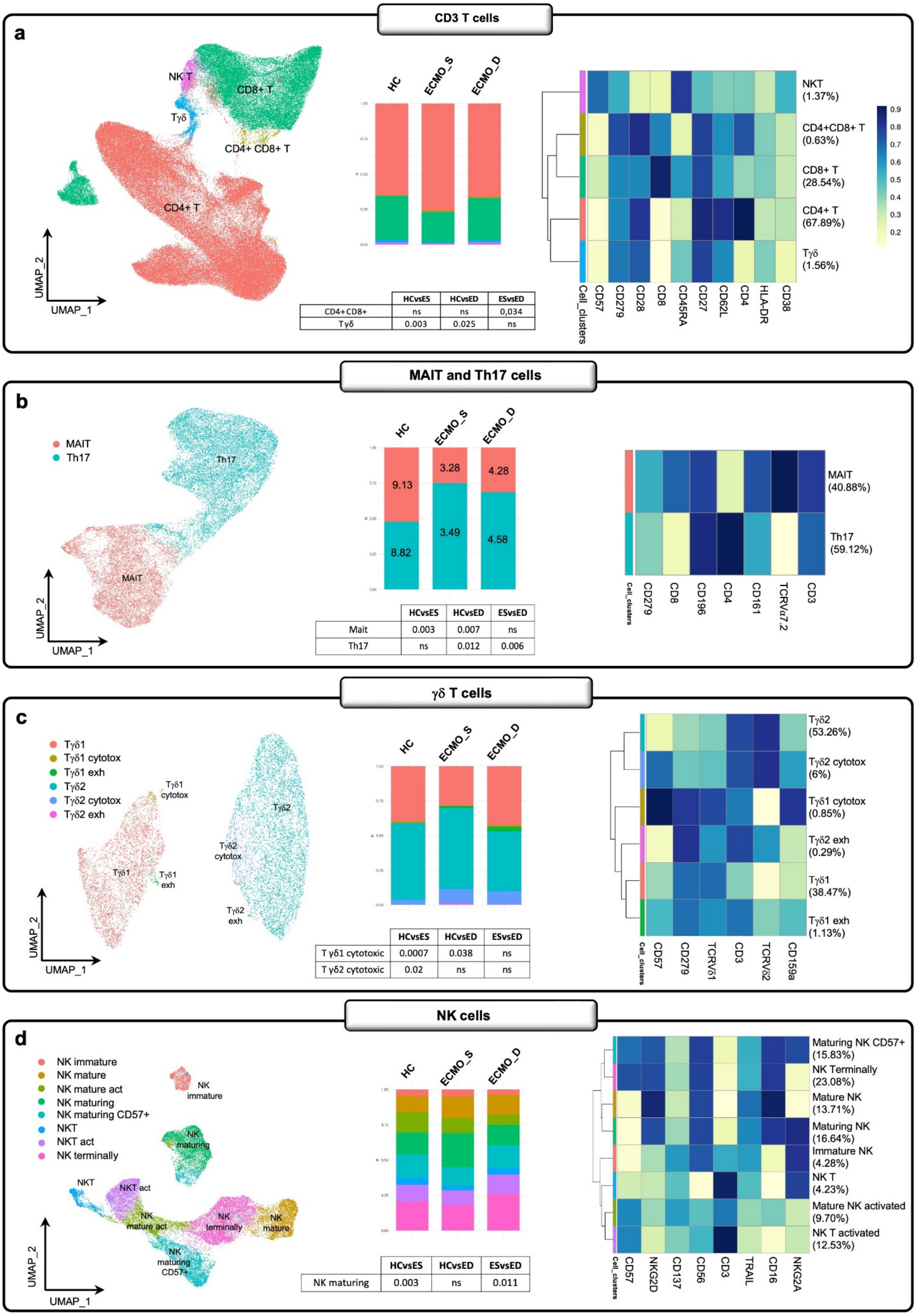

	HCvsES	HCvsED	ESvsED
CD4+CD8+	ns	ns	0,034
Tγδ	0.003	0.025	ns

	HCvsES	HCvsED	ESvsED
Mait	0.003	0.007	ns
Th17	ns	0.012	0.006

	HCvsES	HCvsED	ESvsED
T γδ1 cytotoxic	0.0007	0.038	ns
T γδ2 cytotoxic	0.02	ns	ns

	HCvsES	HCvsED	ESvsED
NK maturing	0.003	ns	0.011

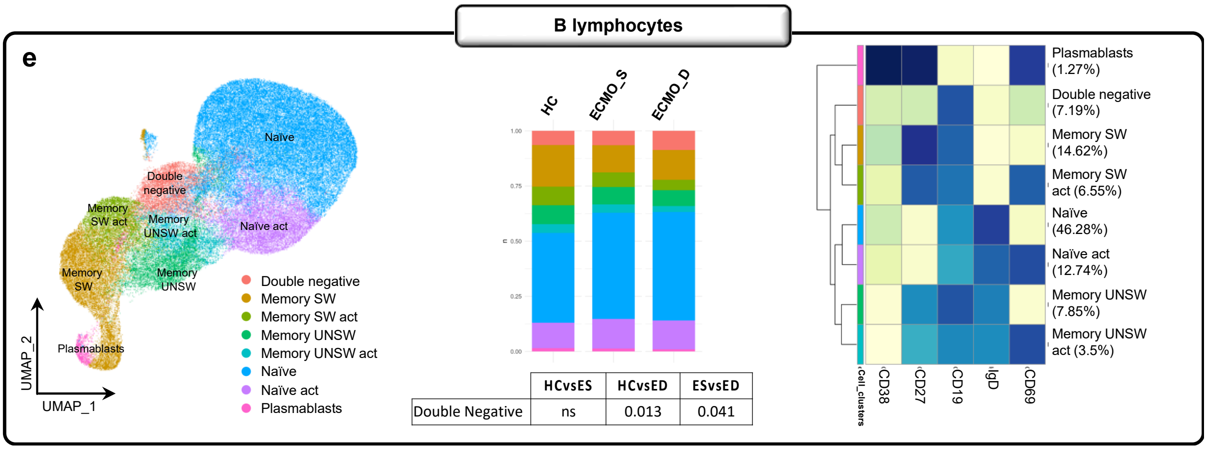

	HCvsES	HCvsED	ESvsED
Double Negative	ns	0.013	0.041

Supplementary Figure S6. **UMAP density plot of immune cell subpopulations.** UMAP density plot shows the distribution of **(a)** CD3^{+} T cells, **(b)** MAIT and Th17 cells, **(c)** T cells, **(d)** NK cells, and **(e)** B lymphocytes in the studied groups. The corresponding panel shows the UMAP density plot, histogram with statistical significance, and biomarkers used for identifying each specific subpopulation. Significances between groups were determined using the Wilcox test. HC: healthy control; ECMO_S: ECMO survived; ECMO_D: ECMO deceased.

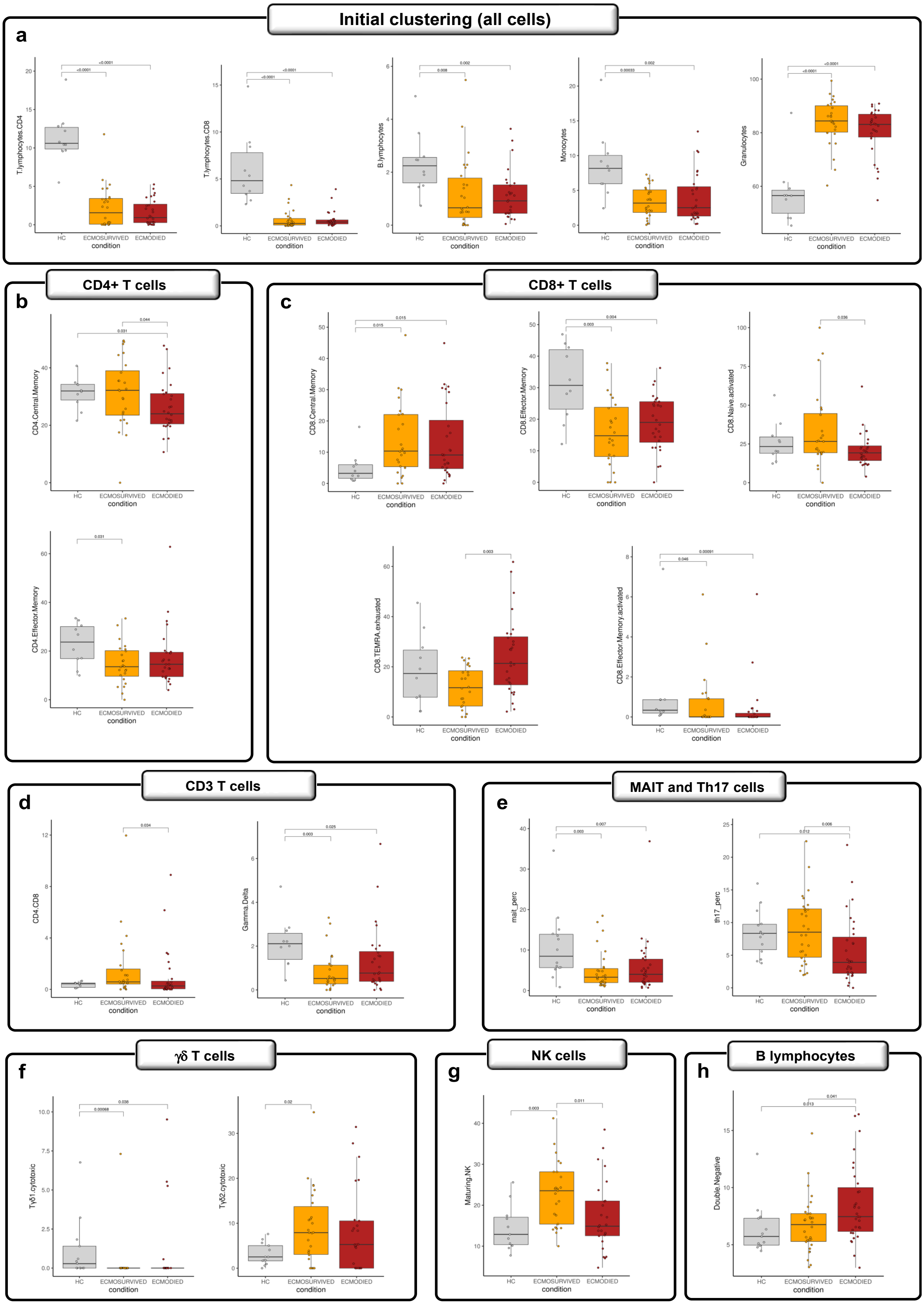

Initial clustering (all cells)
a
T.lymphocytes.CD4
T.lymphocytes.CD8
B.lymphocytes
Monocytes
Granulocytes
HC
ECMOSURVIVED
ECMODIED
condition
CD4+ T cells
b
CD4.Central.Memory
CD4.Effector.Memory
CD8+ T cells
c
CD8.Central.Memory
CD8.Effector.Memory
CD8.Naive.activated
CD8.TEMRA.exhausted
CD8.Effector.Memory.activated
CD3 T cells
d
CD4.CD8
Gamma.Delta
MAIT and Th17 cells
e
mait_perc
th17_perc
γδ T cells
f
Tγδ1.cytotoxic
Tγδ2.cytotoxic
NK cells
g
Maturing.NK
B lymphocytes
h
Double.Negative

Supplementary Figure S7. Immune cell subpopulations. The bar plots show the incidences (% positive cells) of the statistically significant immune cell subpopulations, including **(a)** the initial macro population's distribution ($CD4^+$ and $CD8^+$ T lymphocytes, B lymphocytes, monocytes, and granulocytes), **(b)** the $CD4^+$ T cell subpopulations ($CD4^+$ CM, and $CD4^+$ EM), **(c)** the CD8+ T cells subpopulations ($CD8^+$ CM, $CD8^+$ EM, $CD8^+$ EM $CD279^+$, $CD8^+$ naïve $CD279^+$, and $CD8^+$ TEMRA $CD279^+$), **(d)** the $CD3^+$ T cells subpopulations ($CD4^+CD8^+$ and total $\gamma\delta$ T), **(e)** MAIT and Th17 cell subpopulations, **(f)** $\gamma\delta$ T cell subpopulations (cytotoxic T $\gamma\delta1$, and cytotoxic T $\gamma\delta2$), **(g)** NK cell subpopulations (NK maturing), and **(h)** B lymphocyte subpopulations (double negative). Significances between groups were determined using the Wilcox test. HC: healthy control; ECMO_S: ECMO survived; ECMO_D: ECMO deceased.

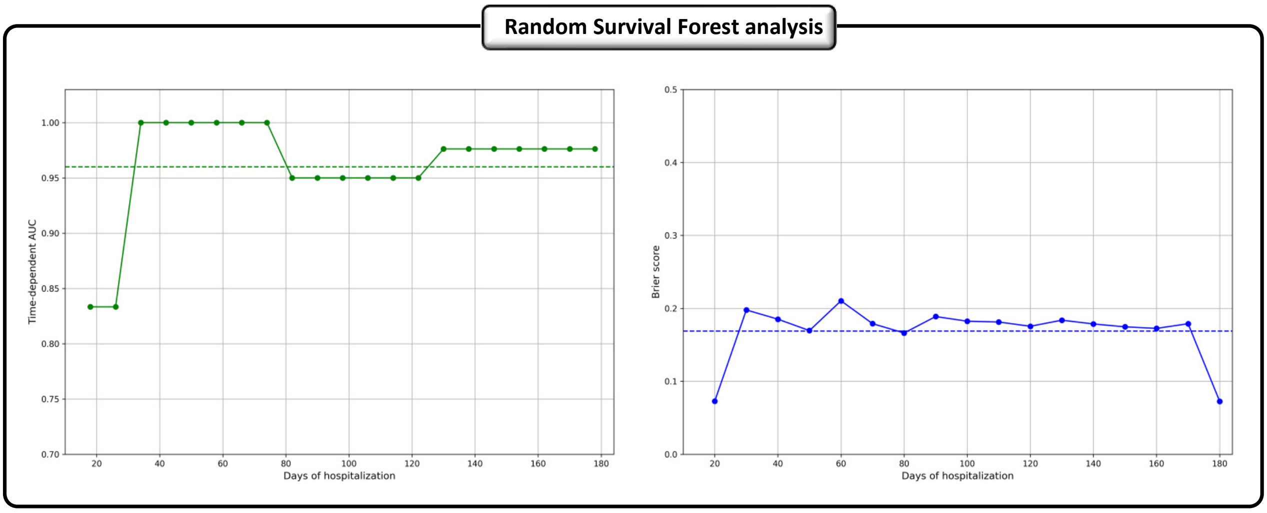

Supplementary Figure S8. **Random survival forest analysis.** Random survival forest model performance statistics over 180 days. Each point is the value at a certain day, while the dotted line denotes the mean value across the entire time window. (**Right**) The time-dependent area under the receiver operating curve (td-AUC) was evaluated at 21 equidistant time points (day 18 to day 180). **(Left)** Brier score was evaluated at 17 equidistant time points (day 18 to day 180).

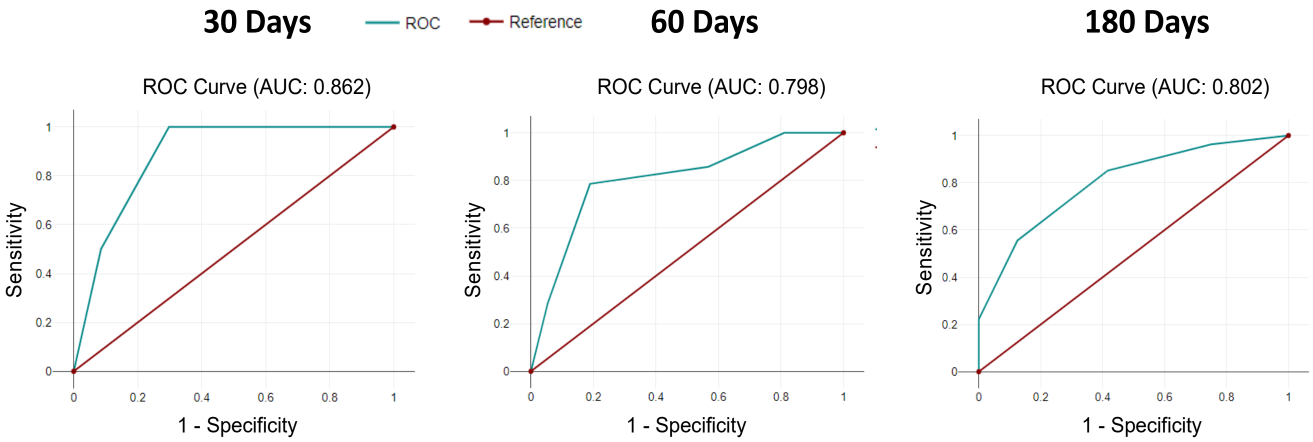

Supplementary Figure S9. ROC analysis. Receiver Operating Characteristic (ROC) curves for evaluating the predictive efficacy of the composite score at various time intervals. The Area Under the Curve (AUC) was calculated to measure the score's performance. **(Left)** ROC curve 30 days after hospitalization, AUC=0.862. **(Center)** ROC curve 60 days after hospitalization, AUC=0.798. **(Right)** ROC curve 180 days after hospitalization, AUC=0.802.

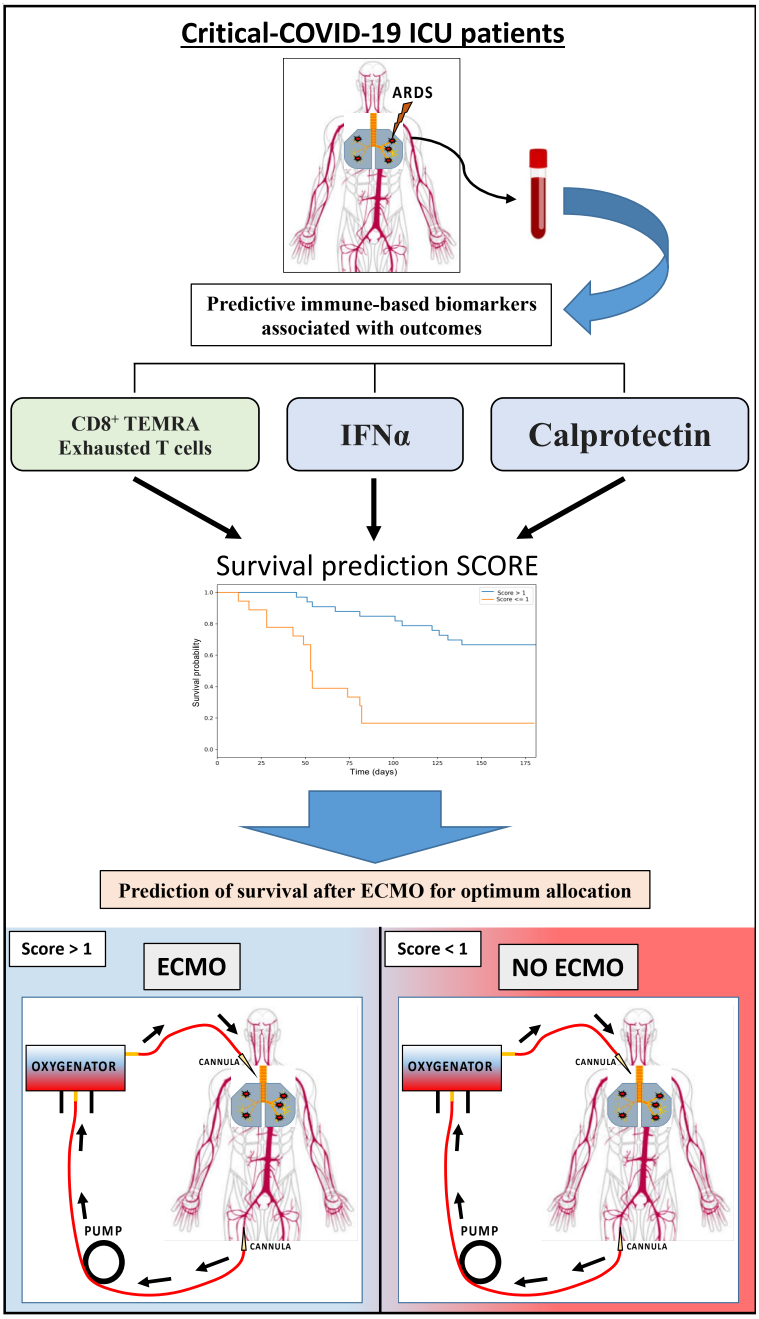

Supplementary Figure S10. Schematic representation of predictor biomarkers as a tool for unsuccessful ECMO treatment.
